# Therapeutic progress and challenges for triple negative breast cancer: targeted therapy and immunotherapy

**DOI:** 10.1186/s43556-022-00071-6

**Published:** 2022-03-04

**Authors:** Ruoning Yang, Yueyi Li, Hang Wang, Taolin Qin, Xiaomeng Yin, Xuelei Ma

**Affiliations:** 1 Department of Biotherapy, State Key Laboratory of Biotherapy,Cancer Center, West China Hospital, 37 Guoxue Alley, Chengdu, 610041 PR China; 2 Department of Breast Surgery, Clinical Research Center for Breast, West China Hospital, Sichuan University, Chengdu, 610041 China; 3 West China Hospital, West China Medical School Sichuan University, Chengdu, PR China

**Keywords:** Triple negative breast cancer, TNBC, Targeted therapy, Immune checkpoint inhibitors, Signaling pathways

## Abstract

Triple negative breast cancer (TNBC) is a subtype of breast cancer, with estrogen receptor, human epidermal growth factor receptor 2 and progesterone receptor negative. TNBC is characterized by high heterogeneity, high rates of metastasis, poor prognosis, and lack of therapeutic targets. Now the treatment of TNBC is still based on surgery and chemotherapy, which is effective only in initial stage but almost useless in advanced stage. And due to the lack of hormone target, hormonal therapies have little beneficial effects. In recent years, signaling pathways and receptor-specific targets have been reported to be effective in TNBC patients under specific clinical conditions. Now targeted therapies have been approved for many other cancers and even other subtypes of breast cancer, but treatment options for TNBC are still limited. Most of TNBC patients showed no response, which may be related to the heterogeneity of TNBC, therefore more effective treatments and predictive biomarkers are needed. In the present review, we summarize potential treatment opinions for TNBC based on the dysregulated receptors and signaling pathways, which play a significant role in multiple stages of TNBC development. We also focus on the application of immunotherapy in TNBC, and summarize the preclinical and clinical trials of therapy for patients with TNBC. We hope to accelerate the research and development of new drugs for TNBC by understanding the relevant mechanisms, and to improve survival.

**Supplementary Information:**

The online version contains supplementary material available at 10.1186/s43556-022-00071-6.

## Introduction

Breast cancer is the most commonly diagnosed cancer in 2020 (11.7% of total cases) and the leading cause of cancer death among women [1]. Triple negative breast cancer (TNBC))is a subtype of breast cancer, which represents 24% of all types of breast cancers [2]. In 2000, the first-generation cDNA microarrays defined basal-like breast cancer as a subtype of breast cancer, which shows low expression level of estrogen receptor (ER) and associated genes, no expression of human epidermal growth factor receptor 2 (HER2) and strong expression of breast basal cell keratins 5/6 and 17 [3]. Then, in 2005, James D et al. defined a group of basal-like breast cancer with ER negative, HER2 negative and progesterone receptor (PR) negative as TNBC by immunohistochemical profiling [4]. In 2011, Lehmann et al. identified six TNBC subtypes, including 2 basal-like (BL1 and BL2), an immunomodulatory (IM), a mesenchymal (M), a mesenchymal stem-like (MSL), and a luminal androgen receptor (LAR) subtype [5].Compared with non-TNBC, TNBC occurs at younger age and shows higher histologic grade and more frequent lymph nodal metastases, meaning aggressive pathology [6]. And due to the lack of receptors, almost all TNBC is insensitive to hormone treatment or anti-HER2 treatment [7]. At present, chemotherapy (anthracyclines and taxanes) is still the dominant treatment for patients with TNBC [5]. Although patients with TNBC have a high rate of clinical response to chemotherapy, they show poor prognosis and high risk of recurrence [8]. And once diagnosed as metastatic TNBC, adjuvant therapy is often ineffective and the median survival time after metastasis is only 13.3 months [8, 9]. Thus, in addition to the development of new applications of existing drugs, there is an urgent need for new drugs and therapies to improve the therapeutic effect of TNBC. With the development of precision therapy, many studies have demonstrated that the targeted therapy has promising value in TNBC. Many abnormal pathways in TNBC have been reported, like Phosphatidylinositol-3-kinase (PI3K)/AKT/ mammalian target of rapamycin (mTOR), the Ras/mitogen-activated protein kinase (MAPK) pathway, the epithelial-mesenchymal transition (EMT) and associated pathways, and so on. Besides, many mutations have been found in TNBC, like germline BRCA1 or BRCA2 mutations. These findings bring new hope for the treatment of TNBC. Besides, target therapy is found to improve the resistance to treatment which often occurs in advanced patients. In the present review, we summarize potential treatment opinions for TNBC based on the dysregulated receptors and signaling pathways, as well as the application of immunotherapy in TNBC, and list related preclinical and clinical trials to provide new ideas and directions for TNBC treatment. We list clinical trials of TNBC with and without results in Supplementary Table 1. And partial of clinical trials with results for patients with TNBC are shown in Table 1.

**Table 1:** Partial clinical trials of targeted agents involving patients with TNBC

Pathway	NCT	Phase	Results		Treatment		Reference
			Group 1	Group 2	Group 1	Group 2	
Combination (Chemotherapy)	NCT02547987	II	pCR: 45.7% (95%CI 36.9–54.7%)		Docetaxel + Carboplatin		[10]
Combination (CDK inhibitor + Paclitaxel)	NCT02779855	I/II	RR: 55%		Talimogene Laherparepvec + Neoadjuvant Chemotherapy (doxorubicin/cyclophosphamide)		[11]
Selective Inhibitor of Nuclear Export	NCT02402764	II	mPFS: 0.92 months (95%CI: 0.62–3.58) mOS: 5.98 months (95%CI:1.68–10.39)		Selinexor		[12]
Combination (CSF-1 inhibitor Chemotherapy)	NCT02435680	II	PFS: 5.6 months (95% CI:4.5–8.7)	PFS: 5.5 months (95% CI: 3.5–7.5)	MCS110 + Chemotherapy (carboplatin + gemcitabine)	Chemotherapy (carboplatin + gemcitabine)	
Chemotherapy	NCT02413320	II	pCR: 54.2%	pCR: 53.8%	Carboplatin + Paclitaxel + Doxorubicin + Cyclophosphamide	Carboplatin + Docetaxel	[13]
Combination (AKT inhibitor + Paclitaxel)	NCT02301988	II	pCR: 17.1% (95% CI, 9.82%-27.25%)	pCR: 13.3% (95% CI, 6.58%-22.86%)	Ipatasertib + Paclitaxel	Placebo + Paclitaxel	[14]
Combination (AKT inhibitor + Paclitaxel)	NCT02423603	II	mPFS: 5.9 months	mPFS: 4.2 months	Paclitaxel + AZD5363	Paclitaxel + Placebo	[15]
Combination (AKT inhibitor + Paclitaxel)	NCT03337724	III	mPFS: 9.3 months (95% CI, 8.0–11.0)	mPFS: 9.3 months (95% CI, 7.2–12.2)	Ipatasertib + Paclitaxel	Placebo + Paclitaxel	[16]
ADC	NCT03106077	II	Stable Disease: 50%		Mirvetuximab Soravtansine		
ADC	NCT02984683	II	TEAEs: 100%	TEAEs: 100%	SAR566658 (90 mg/m^2)	SAR566658 (120 mg/m^2)	
ADC	NCT02078752	I	ORR:8.3 (95% CI 0.2–38.5)		PF-06647263		[17]
ADC	NCT01997333	II	PFS: 2.9 months (95% CI: 2.8–3.5)	PFS: 2.8 months (95% CI: 1.6–3.2)	CDX-011	Capecitabine	
ADC	NCT01631552	I/II	mPFS:5.5 months (95% CI, 4.1- 6.3)		SG		[18]
ADC	NCT02574455	III	mPFS: 5.6 months (95% CI, 4.3–6.3)	mPFS: 1.7 months (95% CI, 1.5–2.6)	SG	Chemotherapy (eribulin/capecitabine/gemcitabine/vinorelbine)	[19]

Clinicaltrials.gov, accessed on November 1, 2021

## Receptor tyrosine kinases and associated pathways

### Receptor tyrosine kinases (RTKs) family

**Fig. 1:**
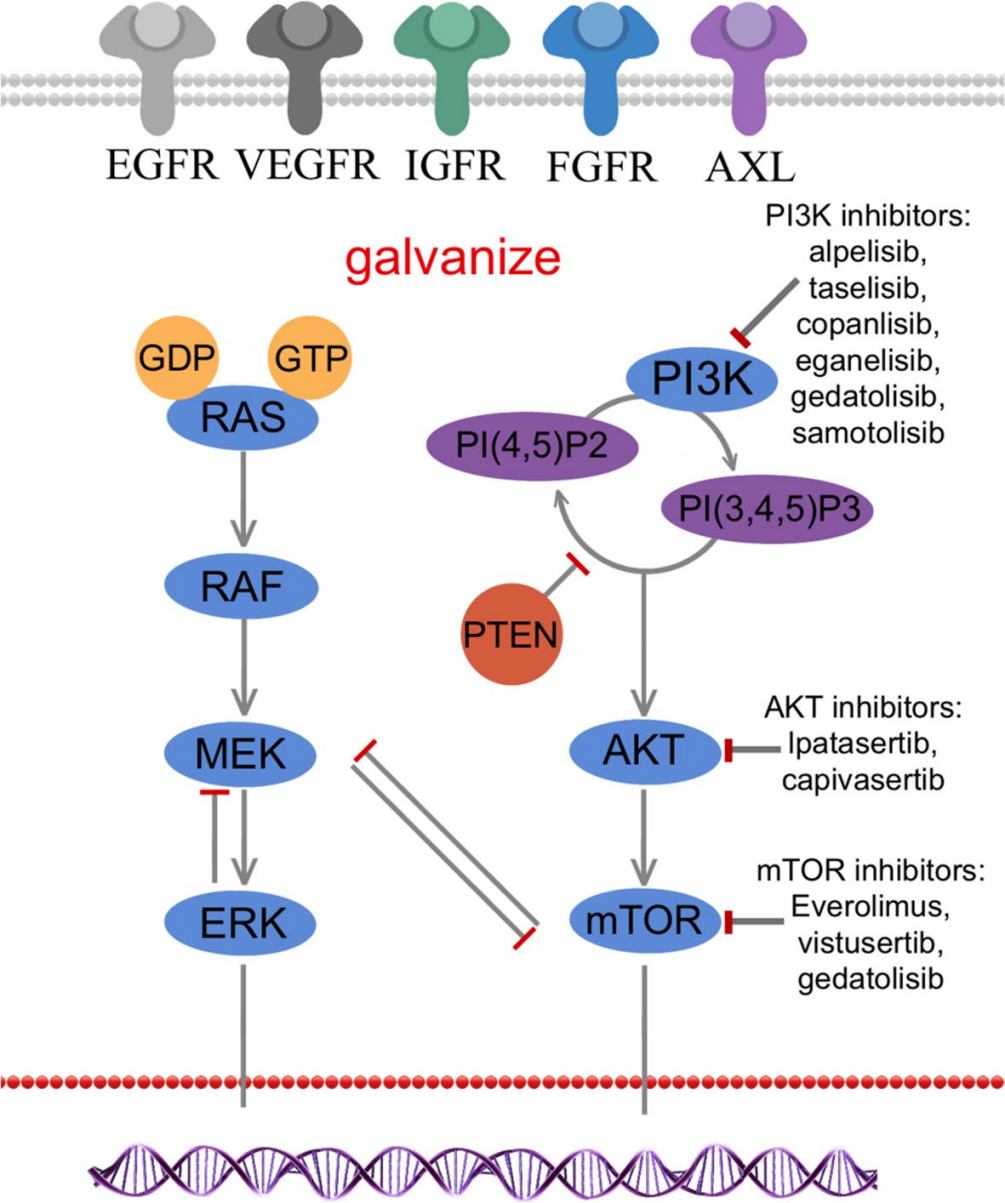
Receptor tyrosine kinases and associated pathways in TNBC. RTKs bind to extracellular ligands, then activated RTKs then activates downstream PI3K/AKT/ mTOR and Ras/MAPK pathway signaling pathways. After Ras family (N-Ras, M-Ras, H-Ras and K-Ras) is activated, downstream Raf, MEK and ERK in turn transfers the signals released from Ras to the nucleus, and finally drives tumor cell proliferation and survival. PI3K is firstly activated by RTKs, and then phosphorylates PIP2 into PIP3, which binds to AKT, and followed by phosphorylation of threonine. And AKT and mTOR are completely activated in turn. PTEN is a negative regulatory phosphatase of PI3K signaling, which can suppress tumor by converting PIP3 to PIP2

RTKs family is a kind of transmembrane enzyme-linked receptor on cell surface, composing of an extracellular ligand-binding region, a single transmembrane helix, a protein tyrosine kinase domain and juxta membrane regulatory regions [20]. There are 58 different kinds of receptors, such as epidermal growth factor receptor (EGFR), vascular endothelial growth factor receptor (VEGFR), insulin-like growth factor receptor (IGFR), fibroblast growth factor receptor (FGFR) and AXL [21]. RTKs binding to ligands to induce the dimerization of the receptors, which then activates the downstream PI3K/AKT/mTOR pathway, RTK/Ras/MAPK pathway and janus kinase/signal transducer and activator of transcription protein family pathway [20, 22]. The conduction processes are showed in Fig. 1. Mutations or disorders of different RTK can drive cancer progression, therefore, it is theoretical plausible to target these RTKs for cancer treatment [23–25]. Now more and more tyrosine kinase inhibitors (TKIs) and anti-TKI antibodies have received approvement from U.S. Food and Drug Administration (FDA) for applications in cancer treatment [20, 22].

TKI is an important targeted drug in tumor therapy and have demonstrated excellent antitumor effects in some cancer. In TNBC patients, the effect of TKIs alone is unclear in the treatment, but it seems unlikely that treatment with tyrosine kinase inhibitors to patients with unselected TNBC would be effective as it is a monotherapy. It needs to be used in combination with other drugs to achieve the desired effect [26]. Lapatinib is the only anti-EGFR agent approved by the FDA for clinical use, and it is recommended to use in combination with chemotherapy or hormone therapy. It was shown to be effective even in trastuzumab-resistant tumors. However, until now the results were frustrating, and no other EGFR inhibitor has made it to clinical use in TNBC [27]. As a common targeted drug, TKIs are less likely to cause toxic effects of traditional chemotherapy such as cumulative bone marrow toxicity, yet it does have a different toxicity profile, as it mainly causes skin and gastrointestinal toxicities, including diarrhea and rashes [28].The biggest challenge for the application of TKIs in clinical practice is drug tolerance, which has been found to be correlated with the overexpressed ALX in resistant tumors [29, 30]. Over 50% of patients with TNBC show overexpression in EGFR overall, indicating that anti-EGFR therapy is promising for TNBC patients with positive EGFR mutations [31]. But early phase clinical trials failed to consistently justify significant effectiveness of anti-EGFR antibody in TNBC patients [26]. Actually, the same result was showed when it combined with routine chemotherapy drug. A meta-analysis shows that cetuximab, the first FDA-approved EGFR-targeted antibody, combined with chemotherapy used in TNBC excelled significant superiority in progression free survival (PFS) over chemotherapy alone [32]. A phase II study compares the pathologic complete response rate, safety and toxicity of cetuximab combined with ixabepilone in TNBC patients (NCT01097642). A triple-combination therapy of ipatasertib, atezolizumab and paclitaxel as treatment for patients with locally advanced or metastatic TNBC is now undergoing phase III trial (NCT04177108). As previously mentioned, the phenomenon of drug resistance is possibly caused by the involvement of AXL in apoptotic cells clearance. Meanwhile AXL is highly expressed in TNBC, and such high expression is significantly associated with lymphovascular invasion, suggesting the potential of targeted-AXL drugs in combination with other targeted agents [33]. Bemcentinib (BGB324) is the first AXL inhibitor applicated in clinical practice [34]. Currently, a phase II trial of bemcentinib combined with pembrolizumab is conducted to examinate the anti-tumor activity and assess the safety of in TNBC patients (NCT03184558). In the future, the researchers should explore the anti-AXL drug in combination with TKIs in TNBC treatment. In conclusion, it is regrettable that there are no drugs targeting RTKs family approved into clinical application for patients with TNBC.

### PI3K/AKT/mTOR signaling pathways

The PI3K/AKT/mTOR pathway is important in chemoresistance and survival of TNBC, involving in cell metabolism, proliferation, migration and survival [35]. PI3K is firstly activated by RTKs, and then phosphorylates 4,5-phosphoinositide (PIP2) into 3,4,5-phosphoinositide (PIP3), which binds to AKT, and followed by phosphorylation of threonine and serine to completely activate AKT [36–38]. In addition, there are other regulatory factors in this cellular pathway. Phosphatase and tension homolog (PTEN), a negative regulatory phosphatase of PI3K signaling, can suppress tumor by converting PIP3 to PIP2 [39]. Through tuberous sclerosis complex 1/2, AKT activates the downstream mTOR, which exists in mTOR complex (mTORC) 1 and mTORC2 respectively [35, 40, 41]. MTORC 1 is linked to lipid synthesis and decomposition, and also regulates growth-stimulatory effects of mTOR [42]. By contrast, mTORC2 can further activate AKT, be involved in cell migration and regulate actin cytoskeleton [43, 44]. Many studies reported that abnormality of the PI3K/PTEN/AKT pathway is presented in more than 25% of TNBC patients, including PIK3CA-activating mutations, PTEN loss, AKT1 activating mutations and mTOR activation [45–48]. Hyperactivation of AKT and mTOR may lead to an unfavorable prognosis of TNBC patients, suggesting that targeting some factors in this pathway is a promising strategy for TNBC treatment [49–51].

Everolimus is a mTORC1 inhibitor, which was approved by the FDA for postmenopausal patients with hormone receptor (HR) + /HER2- advanced breast cancer [52]. The efficacy of everolimus has been confirmed by many studies [53–55]. In a randomized trial, researchers exanimated the safety of the everolimus in patients with hormone-receptor–positive advanced breast cancer. The most common grade 3 or 4 adverse events (AEs) were stomatitis, anemia, dyspnea, hyperglycemia, fatigue, and pneumonitis [52]. Further finding is that the clinical benefit of mTOR inhibitor alone is far from satisfactory, and obviously the combination therapy renders more clinical benefit [56]. Everolimus-carboplatin was reported to be efficacious in metastatic TNBC [57]. Compared with neoadjuvant alone, the combination of everolimus plus cisplatin after neoadjuvant shows lower residual cancer burden [58]. PI3K inhibition would diminish nucleotide pools required for DNA synthesis and S-phase progression, resulting in sensitization to PARP inhibitors in BRCA-proficient TNBC [59]. Based on this, a new, non-chemotherapy treatment option for TNBC with wild-type BRCA might originate from the combination of a PI3K inhibitor and PARP inhibitor. The evaluation over efficacy of gedatolisib, a PI3K and mTOR inhibitor, in combination with talazoparib (PARP inhibitor) in TNBC or BRCA1/2 positive breast cancer with HER2 negative was conducted in a phase II clinical trial. (NCT03911973). And the AEs of gedatolisib have been concerned in other solid tumors. In a multi-arm clinical trial of gedatolisib and irinotecan in advanced sloid cancers, the most common gedatolisib-related AEs were nausea (61.4%), diarrhea (52.3%), vomiting (40.9%), mucosal inflammation/stomatitis (34.1%), decreased appetite (31.8%) and fatigue (29.5%), mostly of grade 1 or 2 [60]. However, there are no studies specifically focusing on AEs of PI3K/AKT/mTOR signaling pathways in patients with TNBC. To sum up, more trials are needed to explore the combinations of PI3K/AKT/mTOR signaling pathways in the future, in addition, how to exert the role of negative regulatory factors in the pathway to achieve anti-tumor effects is also a potential research direction.

### The Ras/ MAPK pathway

Ras family, small GTPases, consisting of N-Ras, M-Ras, H-Ras and K-Ras, can be firstly activated by external stimuli like ligand activation of RTK [61]. Then downstream Raf, MAPK kinase 1 (MEK) and extracellular signal-regulated kinases (ERK) in turn transfers the signals released from Ras to the nucleus, and finally drives cell proliferation and survival [62]. Negative regulation of the pathway is accomplished through the action of DUSP family phosphatases on ERK, the hydrolysis of Ras-associated GTP by NF1, and the negative feedback actions of ERK on both MEK and Raf signaling complexes, among others. The copy number alterations of RAS/MAPK signaling pathways in TNBC was found to be greater than other subtypes of breast cancer, suggesting the connection the pathway activation and TNBC^5^. Excessive positive regulation and lack of negative regulation can both lead to abnormal copy number of the pathway [63]. Inhibitors of MEK is a significant node in the Ras/MAPK pathway, which can inhibit specifically inhibit proliferation of TNBC cell lines [61]. Selumetinib (AZD6244; ARRY-142886), a MEK inhibitor, has shown the ability to inhibit the process of EMT and restrain lung metastasis in animal models of TNBC [64]. A clinical trial in advanced cancer patients with the CI-1040 (an oral MEK inhibitor) shows the usual toxicities of CI-1040 were mild or moderate, like diarrhea, nausea, asthenia, and vomiting [65].Compared with solo therapy, combination therapy with MEK inhibitors seems to be more promising. The combination of CH5126766(RAF/MEK inhibitor) and eribulin was tested to potently inhibit cell growth in TNBC and to suppress the expression of programmed cell death-ligand 1 (PD-L1) [66]. A phase I/II study of AZD2014 (vistusertib, mTOR inhibitors) administered with selumetinib is carrying out a dose-escalation experiment in TNBC patients (NCT02583542).

## Cyclin-dependent kinases (CDKs) 4/6

**Fig. 2:**
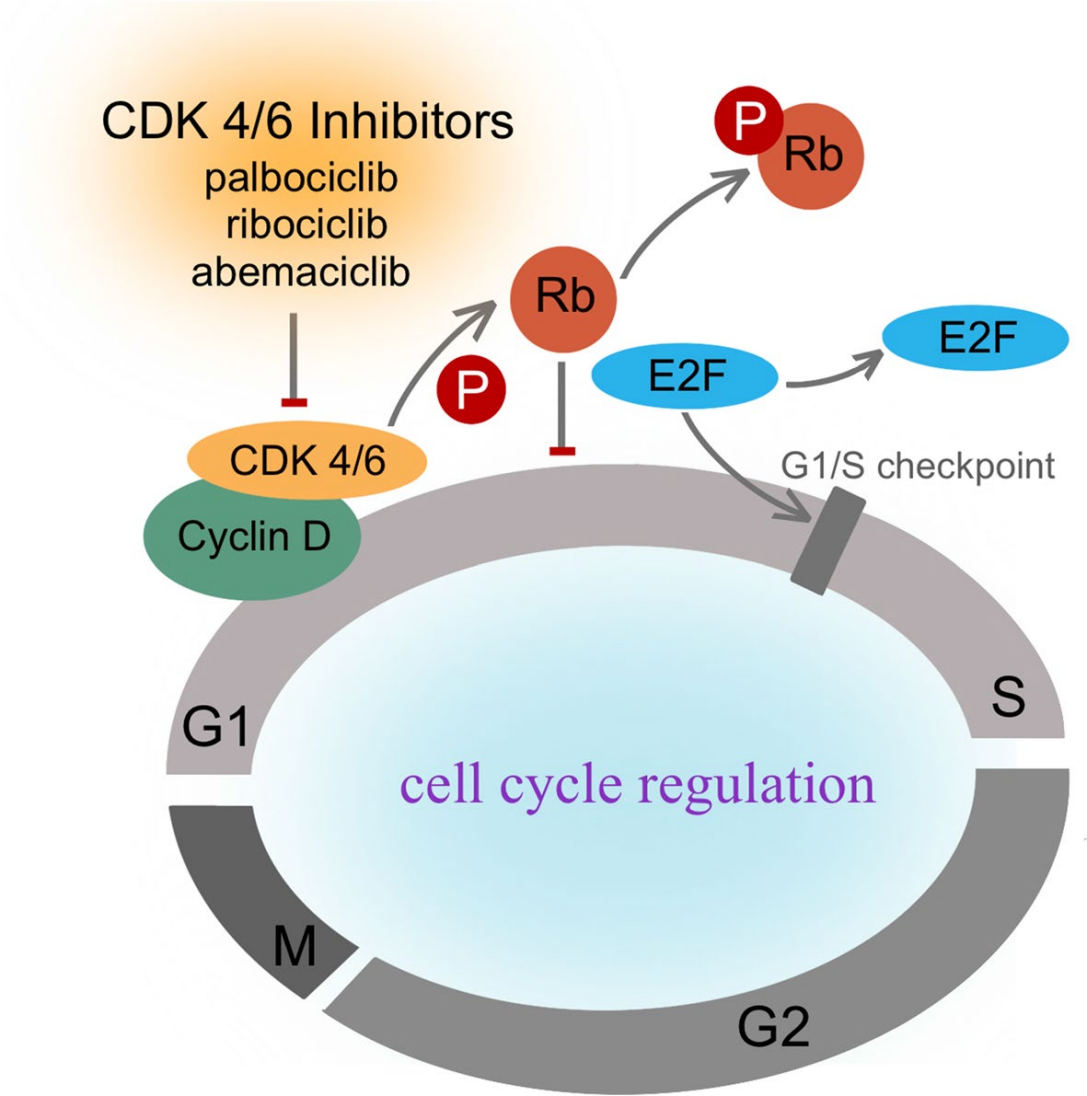
Cyclin-Dependent Kinases 4/6 signaling pathways mediated cell cycle progression in TNBC. CDK 4/6—cyclin D complex gets into the nucleus, and then phosphorylates Rb. Phosphorylated Rb inhibits E2F, and initiates the cell into S phase and drives DNA replication. CDK4/6 inhibitors can inhibit Rb phosphorylation to prevent the proliferation of tumor cells

The mitotic cell cycle is an important process of cell proliferation, depending heavily on continuous activation of several CDKs complexes, while dysregulation of cellular proliferation is considered to be omnipresent in all cancers [67]. Cyclin D, a member of the cyclin family, forms a complex with CDK4/6 kinases to participate in cell cycle progression from the first growth (G1) to the DNA synthesis (S) phase [68]. Cyclin D-CDK4/6 complex gets activated when it gets into the nucleus, and then phosphorylates retinoblastoma protein (Rb). Phosphorylated Rb can inhibit transcription factors like E2F, and initiates the cell into S phase and drives DNA replication [69]. The initial phosphorylation of Rb depends on cyclin D-CDK4/6 complex and the hyperphosphorylation of Rb often leads to the loss of its tumor suppressive function. Therefore, CDK4/6 inhibitors can inhibit Rb phosphorylation to prevent the proliferation of tumor cells. The process of CDK 4/6 signaling pathways mediated cell cycle progression in TNBC is showed in Fig. 2. In addition, CDK5 is an atypical member of the cyclin-dependent kinase family, and its aberrant expression is also related to cell proliferation, DNA damage response, apoptosis, migration and angiogenesis in cancer. Some studies indicated that new post-translational modifications (PTMs) of CDK5 act as molecular switches to control the kinase activity of CDK5 in the cell [11].

Till now, FDA have approved three CDK inhibitors for patients with ER + /HER2 + breast cancer, including palbociclib, ribociclib and abemaciclib [70–72]. Regretfully, no CDK4/6 inhibitor is approved for TNBC and its efficacy in TNBC needs further examination. Genetic analysis of TNBC shows that 20% of TNBC patients have Rb1 loss, 9% of them show cyclin E1 amplification. Lower RB1 expression is considered to be related to the insensitivity to the CDK4/6 inhibitors [45]. Besides, preclinical trials have proven that TNBC was extremely sensitive to CDK4/6 inhibitors in vitro and vivo, especially for luminal androgen receptor (LAR) subtype [73, 74], suggesting that there may be yet-undiscovered mechanisms. There are many ongoing studies on CDK4/6 inhibitors in TNBC. Now, a phase II clinical trial is evaluting the efficacy and safety of aemaciclib for patients with Rb + TNBC. (NCT03130439). Besides, CDKs link cell proliferation with other signaling pathways, making the combination therapy also worth exploring. For example, CDK4/6 can regulate T cells to enhance immune function and inhibit the cytotoxicity of chemotherapy [67]. What’s more, a phase I trial confirmed that palbociclib combined with paclitaxel is safe and free of additive toxicity for patients with Rb + breast cancer (NCT01320592). And the most common palbociclib-related AE in this trial was neutropenia, which was also the most common grade 3/4 event, and other AEs may have relations with Paclitaxel therapy [11].Trilaciclib, a CDK 4/6 inhibitor, is in a clinical trial to exam its safety and efficacy when administered prior to chemotherapy in patients receiving first- or second-line treatment for locally advanced unresectable or metastatic TNBC (NCT04799249). And the expression of Rb was found to be highly related to androgen receptor (AR) expression in TNBC patients [75]. Targeting AR in TNBC has shown promising result and the combination of CDK 4/6 inhibitors and AR antagonists is also beneficial [75].

## Notching signaling

The highly conserved Notch signaling is involved in angiogenesis, tumor growth, invasion and metastasis, even leading to the poor prognosis, resistance to treatments, and relapse of TNBC [76, 77]. Notch receptors on the surface of one cell bind to ligands of a neighboring cell, thus activate Notching pathways. As a single transmembrane protein, Notch ligand is composed of two parts: a typical extracellular DSL domain mediating receptor binding and multiple EGF-like repeats. Till now, 5 Notch ligands, Delta-like (Dll) 1, 3, 4, and Jagged (JAG)1, 2, respectively and 4 Notch transmembrane receptors, Notch 1–4, have been found [78]. The Notch ligand–receptor complex is hydrolyzed by the ADAM17/TACE metalloprotease and γ-secretase in turn, releasing the intracellular domain of Notch into the nucleus. Then the downstream gene transcription is activated, including cell-cycle regulators, transcription factors, and regulators of angiogenesis and apoptosis [78–80]. In addition, many other signaling pathway may affect the Notch signaling pathway, for instance, VEGFR3、ER and HEY pathways [81]. Studies have found that Notch signaling pathway is overactivated in TNBC [76, 82]. Thus, targeting Notch signaling may be a promising treatment strategy. Although no FDA-approved therapy has existed for the Notch signaling pathway so far, there are still many studies exploring how to suppress Notch signaling at different levels of the cascade, among which the most promising is aspartyl protease inhibitors or γ-secretase inhibitors (GSIs), and several GSIs are already in clinical trials. For instance, as a powerful and selective small molecule GSIs, RO4929097 was found to be well-tolerated when given in intermittent or daily dosing [83]. The phase II clinical trial about RO4929097 in treating patients with advanced, metastatic, or recurrent TNBC has ended (NCT01151449). However, the GSIs are prone to AEs like diarrhea, affecting the digestive, circulation, hematological and other systems due to poor bioavailability and off-target side effects [84]. Dose-limiting intestinal toxicity is another disadvantage, which is also the main restriction of its clinical application [85]. To reduce the drug tocicity, intermittent administration has been proposed, but whether this affects the drug effect remains to be unclear [86]. In addition, great tolerance was observed in a clinical trial of an oral selective RO4929097 in combination with neoadjuvant chemotherapy in TNBC (NCT01238133), indicating that GSIs are effective in combination therapies [83]. CB-103 is a novel synthetic modulator of the Notch pathway, which seems to be more suitable for clinical application as it has no serious side effects or cytotoxicity compared with RO4929097. It is currently in clinical development. A phase I/II, dose escalation study investigates the safety, tolerability and preliminary efficacy of CB-103 in adult patients with advanced or metastatic solid tumors and hematological malignancies in “Recruiting” stage (NCT03422679).

## Poly-(ADP)-Ribose polymerase (PARPi)

**Fig. 3:**
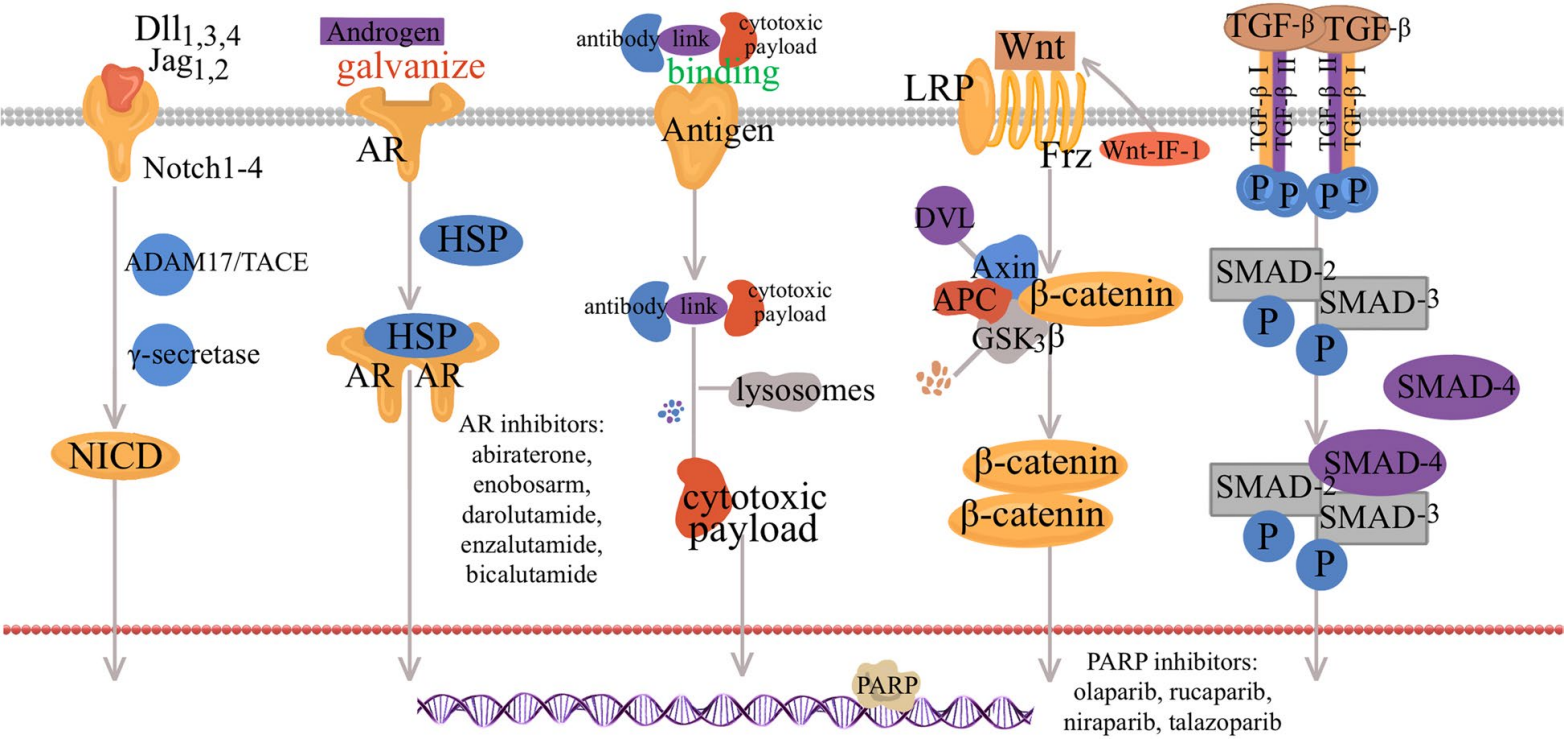
Partial Signaling pathways and potential targets for the treatment of TNBC. PARP is a DNA repair enzyme, which can promote the repair of single strand DNA breaks. PARP inhibitors can suppress DNA repair resulting in cytotoxicity. Notch receptors (Notch 1–4) on the surface of one cell bind to ligands ( Dll 1, 3, 4, and JAG 1, 2), thus activate Notching pathways. The Notch ligand–receptor complex is hydrolyzed by the ADAM17/TACE metalloprotease and γ-secretase in turn, releasing the intracellular domain of Notch into the nucleus. AR bind to endogenous androgens, and then form a homodimer. Before homodimer, AR is bound to HSP. Subsequently, the homodimer moves into to the nucleus and activates target gene transcription, regulating cell proliferation. ADCs consist of a highly efficient cytotoxic payload, an antibody and a linker connecting the former two components. ADC binds to target antigens on tumor cell surface and then enter the target cell via receptor-mediated endocytosis. After entering the cell, ADCs are degraded by lysosomes, in which the linker cleaves, leading to the release of payloads into cytoplasm. When the Wnt signaling is on, Wnt/β-catenin signaling pathway is activated from the combination of lipid-modified Wnts and the receptor complex which is composed of Fzds and LRP5/6. Dvl inhibit the destruction complex, inhibiting the β-catenin proteasomal degradation. therefore, the β-catenin enter into the nucleus. After entering into the nucleus, β-catenin binds to T cell factor and LEF families and regulates Wnt target gene expression. When Wnt ligands are absent in the cytoplasm, β-catenin is sequestered by the destruction complex (APC, GSK-3β and CK1α), and then phosphorylated by CK1α and GSK-3β in turn. TGF-β is excreted by cells and then successively binds to TβRII and TβRI on the surface of cell. During this process, two TβRI and two TβRII molecules form a heterotetrametric complex, further causing the phosphorylation and activation of TβRI. After that, the activated complex phosphorylates Smad2 and Smad3 in sequence, which bind to SMAD4 to form SMAD trimer complex. Finally, the complex translocates into nuclei and promotes target gene transcription

Breaks in DNA double-strand is a common DNA damage associated with tumorigenesis and BRCA 1 and BRCA 2 can repair the breaks in normal cell [87, 88]. More than 15% patients with TNBC have BRCA 1 or 2 mutations, and patients with TNBC and BRCA2 mutation have similarities in clinical and pathological feature [89, 90]. There is evidence that PARP, a DNA repair enzyme, can promote the repair of single strand DNA breaks, which is a crucial way to repair when BRCA mutations occur. The disruption of PARP can lead to delayed repair and increased sensitivity to agents that induce base alkylation or DNA strand breaks [91]. This process is showed in Fig. 3. PARP inhibitors (PARPis) can suppress DNA repair through either poly-ADP ribosylation or the homologous recombination pathway, resulting in cytotoxicity, which makes great progress in TNBC patients with BRCA 1/2 mutations [92]. There are so far four common PARPis that have been developed, including olaparib, rucaparib, niraparib, and talazoparib, respectively [93]. In 2018, FDA approved olaparib and talazoparib for breast cancer with BRCA 1/2 mutations and HER2 negative [94]. The benefit of single agent olaparib in metastatic breast cancer patients with BRCA 1/2 mutations has been tested in a phase III study (NCT02000622). In this trial, patients receiving olaparib monotherapy showed a 2.8-months-longer median PFS (mPFS) and a 42%-lower-risk of disease progression or death than receiving chemotherapy [95]. In the EMBRACA phase III trial that evaluated breast cancer patients with HER2 negative and germline BRCA1/2 mutations (NCT01945775), patients with talazoparib showed statistically overall improvements and significant delay in time to definitive clinically meaningful deterioration compared with those received chemotherapy (PFS: 8.6 vs. 5.6 months; hazard ratio = 0.54) [96]. The remaining two PARPis, rucaparib and niraparib, are still in clinical trials. However, acquired resistance to PARPis in TNBC patients is an evitable problem and limits its use in clinical application [93]. To solve this, further molecular and clinical studies should be conducted to reveal the mechanisms of PARPi-resistance. Several researchers have proposed combination therapy with PARPis to overcome the resistance, which shows promising clinical prospects. For example, PARPis show better therapeutic effects when used in combination with platinum [97]. A phase II/III trial evaluates the safety and efficacy of the combination of platinum and olaparib in patients with TNBC (NCT03150576). A phase I/II study tests the safety and efficacy in TNBC patients with niraparib and pembrolizumab (NCT02657889) [98]. At present, except for the researches on combination therapy, researchers also have also focused on the clinical benefit of PARPis in TNBC patients without BRCA 1/2 mutations. In addition, PARPi resistance is still the main problem limiting the clinic application of PARPis. Further molecular and clinical studies should be conducted to reveal the mechanisms of PARPi-resistance.

## Epithelial-to-mesenchymal transition and associated pathways

### Wnt/β-Catenin signaling pathway

As the canonical Wnt signaling, Wnt/β-catenin signaling pathway is a highly conserved signaling pathway, associated with the progression of various tumors [99]. The upregulation of the Wnt/β-catenin signaling pathway in patients with TNBC is found to have connections with the process of tumor proliferation, metastasis and the resistance to anticancer agents [100]. When Wnt ligands are absent in the cytoplasm, β-catenin is sequestered by the destruction complex (composing of adenomatous polyposis coli (APC), Axin, glycogen synthase kinase 3β (GSK-3β) and casein kinase 1α (CK1α)), and then phosphorylated by CK1α and GSK-3β in turn [101–104]. Cullin1 and F-box protein β-TrCP can promote the ubiquitination of the destruction complex, leading to the degradation of β-catenin and the transcriptional repression of Wnt target genes [104].

When the destruction complex is inhibited by Dishevelled (Dvl), the β-catenin proteasomal degradation is disrupted, therefore, the β-catenin enter into the nucleus [105]. After entering into the nucleus, β-catenin binds to T cell factor and LEF families and regulates Wnt target gene expression [106].When the Wnt signaling is on, Wnt/β-catenin signaling pathway is activated from the combination of lipid-modified Wnts and the receptor complex which is composed of Frizzleds (Fzds) and low-density lipoprotein receptor-related protein 5/6 (LRP5/6). This process is showed in Fig. 3. So far, Fzd7 and LRP6 have been found to be upregulated in TNBC, thus, preventing Wnts from binding to the receptors can be beneficial to targeted therapy for TNBC [107–109]. For example, endogenous inhibitors like Wnt inhibitory factor 1 (WIF-1) can interact with Wnts to directly inhibit the pathway [110]. To our delight, deregulation of Wnt/β-catenin signaling is found to be crucial in the development of the resistance to anti-cancer agents [99]. A series of agents for Wnt signaling pathway in TNBC have been developed through the past couple of decades, such as inhibition of Fzds or Dvl, inhibition or degradation of β-catenin and so on. Besides, it has been proved that targeting Wnt pathways can improve the resistance to carboplatin, thereby the combination of Wnt inhibitors and carboplatin can be an option in treatment of TNBC [111]. However, there are no available Wnt-targeted inhibitors applicated in clinical practice so far.

### TGF-β /Smad signaling pathway

TGF-β signaling pathway plays a biphasic part in tumor progression, where TGF-β serves as a tumor suppressor at the early stage and a tumor promoter at advanced stages [112]. There are three TGF-β subtypes TGF-β1, TGF-β2, and TGF-β3. As a secreted polypeptide, TGF-β is excreted by cells and then successively binds to TGF-β receptors on the surface of cells [113], including TGF-β receptor type II (TβRII) and I (TβRI). During this process, two TβRI and two TβRII molecules form a heterotetrametric complex, further causing the phosphorylation and activation of TβRI [114]. After that, the activated complex phosphorylates Smad2 and Smad3 in sequence, which bind to SMAD4 to form SMAD trimer complex. Finally, the complex translocates into nuclei and promotes target gene transcription [115, 116]. When this pathway is overactivated, for instance, the overexpression of Smad-2 or Smad-3, the EMT can be induced inappropriately, mediating cancer metastasis [117, 118]. This process is illustrated in Fig. 3. Compared with non-TNBC cells, the level of TGF-β1 mRNA, cell invasiveness and protein expression were evaluated to be higher in TNBC cells, which may have connections with the invasion and migration of TNBC [119]. Consequently, the motility and tumorigenicity of TNBC cells can be suppressed by the inhibition of TGF-β/Smad signaling pathway. In an in-vitro cell model, zerumbone is found to induce the phosphorylation of Smad3, thereby suppressing the tumorigenicity of TNBC cells [119]. Another drug, LY2109761, a dual TGF-β receptor I/II inhibitor, suppresses the TNBC cell migration and prevents the occurrence of metastasis [119, 120]. Moreover, metformin is confirmed to block endogenous activation of Smad2 and Smad3 to inhibit the TGF-β/Smad signaling pathway. Either metformin alone or combining with LY2197299 (galunisertib), a selective TGF-β receptor I-kinase inhibitor, can attenuate TGF-β-induced proliferation [121, 122]. This discovery may provide a new option for TNBC patients clinically. Additionally, studies on TNBC cells suggest cancer stem-like cells (CSC) promote the process of chemotherapy-resistance and recurrence [123], and targeting TGF-β signaling pathways can decrease the CSCs population in TNBC patients receiving chemotherapy [114]. For instance, LY2197299 has been demonstrated to inhibit the development of CSCs, suggesting the combination of chemotherapy and TGF-β -targeted agents as a potentially therapeutic option in TNBC [123]. Besides, a phase I trial is studying the best dose and side effects of M7824, an anti-PD-L1/TGF-β antibody, when given together with eribulin mesylatein in patients with metastatic TNBC (NCT03579472). For future investigations, whether TGF-β inhibitors in conjunction with conventional treatment can improve the curative effect remains to be determined. Whether assessing CSC population is needed to guide treatment also needs further discussion.

## Androgen receptors

It has been indicated that AR is expressed in about 35% of TNBC and plays an important role as a potential therapeutic target [124]. According to gene expression profiles, TNBC with AR signaling is called a LAR subtype [5]. AR is a member of the steroid hormone receptor family, which is usually bound to heat shock proteins before homodimer. Therefore, AR can bind to the ligand such as endogenous androgens, and then form a homodimer. Subsequently, the homodimer moves into to the nucleus and activates target gene transcription, regulating cell proliferation [125, 126]. This process is showed in Fig. 3. Base on the above characteristics, anti-androgens, selective androgen receptor modulators and 7-hydroxytestosterone are effective for patients with AR positive breast cancer, including AR positive TNBC. Bicalutamide is the first AR antagonist which was clinically evaluated in 2013. The phase II study exploring bicalutamide has proved that antiandrogen therapy is effective in treating patients with AR positive breast cancer [127]. Further, the antitumor activity and safety of enzalutamide in patients with AR positive TNBC has been assessed in another phase II trial (NCT01889238). The results showed that the clinical benefit rate at 16 weeks was 25%, and the median overall survival was 12.7 months, meaning considerable beneficial therapeutic efficiency. And the only treatment-related grade 3 or higher AE was fatigue with an incidence > 2% [128]. Except for the above two kinds of AR antagonist, a past study found that LAR cell lines were especially sensitive to NVP-BEZ235, a dual PI3K/mTOR inhibitor, which can be explained by PIK3CA-activating mutations in all LAR cell lines [5]. For TNBC patients with AR positive, they benefit not only from monotherapy but also combination therapy. A study shows that compared with AR negative TNBC, activated PIK3CA mutations are abundant in TNBC with AR positive, and the combination of PI3K inhibitors and AR antagonist can significantly inhibit the growth and viability of LAR cell line models [129]. Moreover, it is proven that the combination of bicalutamide with the EGFR inhibitors more effectively decreases the expression of AR compared with each agent given alone, and suppress cell proliferation of tumor [129]. Therefore, recent topic has been focused on the combination therapy of AR pathway inhibition in TNBC. It’s probably also worth noting that how to define AR positive. Now patients selected for clinical trials are mainly LAR subtype defined by gene expression profiling. So, can we precisely guide therapy based on AR expression levels?We need more evidence to explore.

## Antibody–drug conjugates (ADC)

ADCs` are immunoconjugate agents with specificity and the high potency, which can deliver chemotherapy drugs to cancer cells precisely. ADCs consist of three components, a highly efficient cytotoxic payload, an antibody and a linker connecting the former two components [130]. In the process of transport, ADC binds to target antigens on tumor cell surface and then enter the target cell via receptor-mediated endocytosis. After entering the cell, ADCs are degraded by lysosomes, in which the linker cleaves, leading to the release of payloads into cytoplasm. This process is illustrated in Fig. 3. Regarding the efficacy in the treatment of solid tumors, ADCs have shown great clinical prospect, they are rarely used in TNBC patients because of lack of effective and appropriate targets [131]. Till now, there have been three ADCs approved by FDA for breast cancer, two of which, ado-trastuzumab emtansine and fam-trastuzumab deruxtecan, target HER2 [132]. Another one, sacituzumab govitecan (SG), is approved for patients with metastatic TNBC who have received at least 2 kinds of therapy before. SG, composed of an anti–Trop-2 antibody and active metabolite of irinotecan, targets the antigen trophoblast cell-surface antigen 2 (Trop-2), which is a glycoprotein expressed by many solid cancers [133]. Preclinical trials show that Trop-2 expresses in all breast cancer subtypes, particularly in TNBC [134]. Furthermore, in a clinical trial in patients with metastasis TNBC, SG reveals manageable safety, with 33% objective response rates and a mPFS of 5.5 months [135]. The subsequent phase III ASCENT trial confirms improvements in both PFS and OS, which accelerates US FDA approval of SG [136]. Another phase III study, SASCIA, assesses the therapeutic effect of SG in primary HER2 negative breast cancer patients (HR + or TNBC) with high relapse risk after standard neoadjuvant treatment (NCT04595565). In addition, more ADCs targeting novel antigens in TNBC patients are in clinical trials, including LIV-1, receptor tyrosine kinase-like orphan receptor 1 (ROR1) and ROR2.

Compared with conventional chemotherapy, ADC can preciously target the tumor cells, which provide a broad therapeutic window. For TNBC with high heterogeneity, ADC is more suitable for combination therapy with other targeted agents to enhance synergy. Thus, it’s promising research to explore more combination strategy. Besides, more attention should be paid to its toxicities. In addition to off-target effects, AEs are related to payload effectiveness. ADCs combined with non-overlapping toxic immune checkpoint inhibitors (ICIs) may be a potential option.

## Immune checkpoint inhibitors (ICIs)

**Fig. 4:**
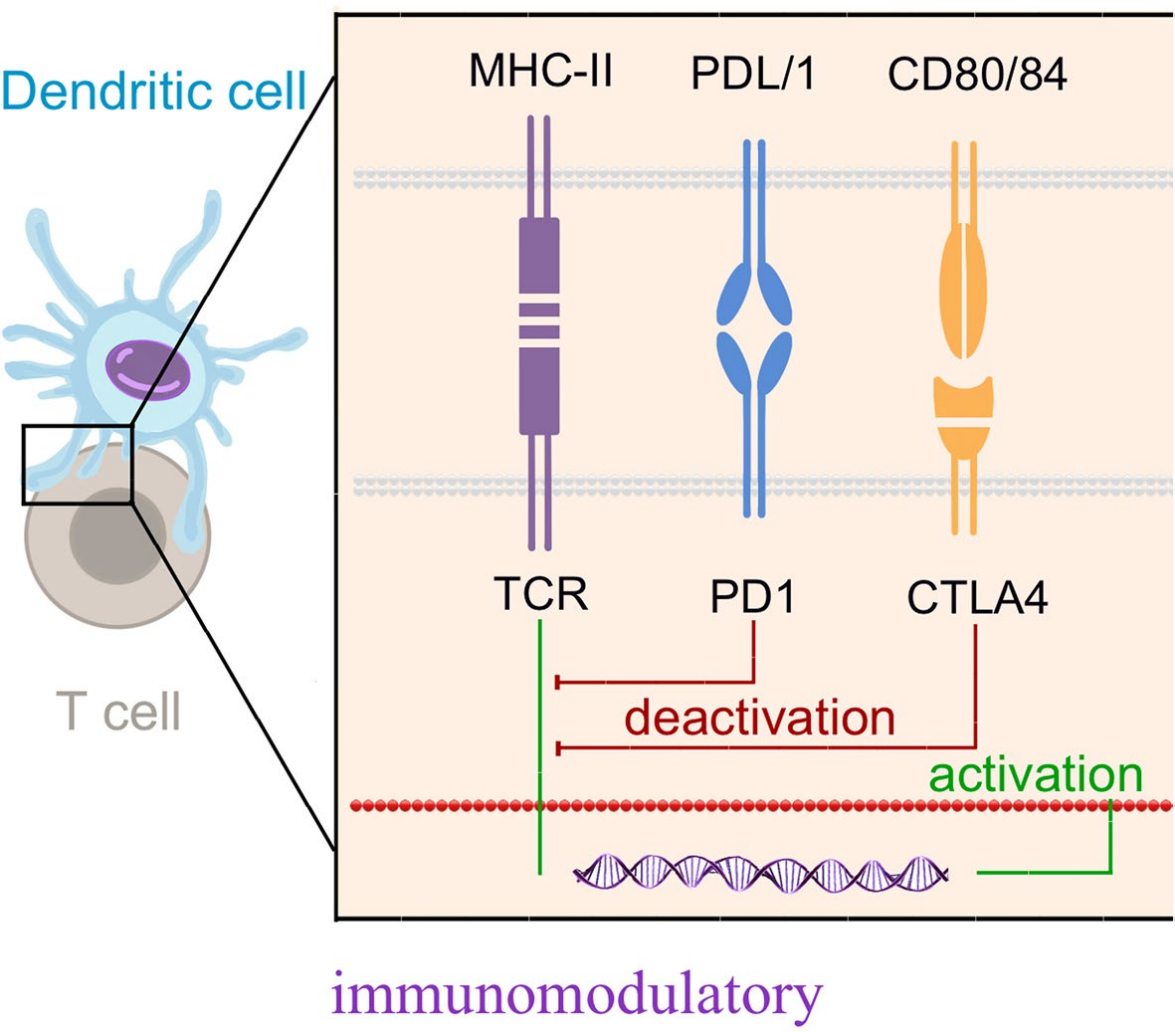
Immune checkpoint inhibitors for the treatment of TNBC. Immune checkpoints limit the anti-tumor autoimmune response by inhibiting effector T lymphocytes. PD-L1 expressing in the tumor cells binds to PD-1 which is on the surface of T cell and prevents T cell activation. The tumor-infiltrating lymphocytes also can express highly PD-1, and both of them are increased in TNBC. ICIs can enhance the anti-tumor immune responses to kill tumor cell

Immune checkpoints, most notably cytotoxic T-lymphocyte-associated protein 4 (CTLA-4) and programmed cell death protein 1 (PD-1), inhibit effector T lymphocytes to limit the anti-tumor autoimmune response [137]. PD-L1 expressing in the tumor cells binds to PD-1 which is on the surface of T cell and prevents T cell activation [138]. The tumor-infiltrating lymphocytes also can express highly PD-1, and both of them are increased in TNBC [139]. Therefore, ICIs can enhance the anti-tumor immune responses to kill tumor cells [140]. These processes are illustrated in Fig. 4. ICIs including monoclonal antibodies against PD-1 (pembrolizumab, nivolumab), PD-L1 (atezolizumab, durvalumab, avelumab), and CTLA-4 (ipilimumab) have shown promising results in treating many types of cancer. Nowadays, there have been two ICIs approved by the FDA. One is Atezolizumab in 2019, which is approved in combination with nab-paclitaxel to treat unresectable locally advanced or metastatic TNBC patients with PD-L1 positive in 2019 [141]. In a phase III clinical trial (KEYNOTE-355), the other ICI, pembrolizumab plus chemotherapy distinctly improved the PFS of patients with metastatic TNBC, compared with placebo plus chemotherapy [142]. And therefore, Pembrolizumab, was approved in 2020 to treat metastatic TNBC patients with PD-L1-expressing in combination with chemotherapy [143]. In addition to combining chemotherapy, the combination of ICIs and targeted agents in the treatment of TNBC also has great clinical potential. For example, PARPis can lead to cell death through inhibiting the repair of DNA damage. Surprisingly, repressed PARP enhances PD-L1 expression in TNBC cells, creating conditions for ICIs to target [144]. In a clinical trial, niraparib-pembrolizumab shows potential anti-tumor activity for patients with advanced or metastatic TNBC, and significantly higher response rates in patients with BRCA mutations (NCT02657889) [98]. Furthermore, many other ICIs are also being studied in clinical trials and promising predictive biomarkers for immunotherapy in TNBC are also very potential in clinical practice.

Combination with other therapies or factors can also augment the efficacy of ICIS. The first of the therapy is Dysregulated Tumor Vasculature. When the tumor expands to a certain extent, tumor cells within the tumor core become increasingly hypoxic, and expresses the Angiogenic Growth Factors, which is associated with the expression of hypoxic induced transcription factors [145, 146]. Under the hypoxia conditions of tumor microenvironment, the metabolic demand balance of angiogenesis and surrounding tissue is destroyed, causes angiogenesis [146]. In the IMbrave150phase III clinical trial (NCT03434379), the anti-PD-1 reagent, combined with an anti-angiogenic agent, had a clinically prominent amelioration in mPFS, compared with the protein kinase inhibitor [147]. The other one is Interleukin-8 and CXCR1 / CXCR2. IL-8 can bind to CXCR1 and CXCR2 G-Protein coupled receptors on granulocytes, monocytes, and endothelial cells [148, 149]. Some research indicated that anti-CXCR2 monoclonal antibody caused remarkable tumor-resistance activity, even after delayed anti-PD-1 treatment [150]. The Phase I Trial for Humax-IL8 (NCT 02,536,469) indicates that serum IL-8 levels have decreased significantly in patients who use IL-8 as a prognostic biomarker to inhibit anti-PD-1 checkpoints. This indicates that IL-8 suppression can be regarded as potential candidates for ICI combination treatment [151]. Further, Cluster of differentiation 73, normally expressed on Treg cells, is an ectonucleotidase that dephosphorylates extracellular AMP to adenosine [152]. It can promote the adhesion of lymphocytes restrain the migration of lymphocytes and reduce lymph nodes [153]. We summarize partial of clinical trials with results on ICIs in Table 2.

**Table 2:** Partial clinical trials of ICIs involving patients with TNBC

Pathway	NCT	Phase	Results		Treatment		Reference
			Group 1	Group 2	Group 1	Group 2	
ICIs (Anti PD-1)	NCT03125902	III	mPFS: 6.0 months mOS: 22.1 months ORR: 63% (95% CI, 56%-70%)	mPFS: 5.7 months mOS: 28.3 months ORR: 55% (95% CI, 45%-65%)	Paclitaxel + Atezolizumab	Paclitaxel + Placebo	[154]
ICIs (Anti PD-1)	NCT03197935	III	pCR: 58% treatment-related serious adverse events: 23%	pCR: 41% treatment-related serious adverse events: 16%	Atezolizumab + Chemotherapy (nab-paclitaxel + doxorubicin + cyclophosphamide)	Placebo + Chemotherapy (nab-paclitaxel + doxorubicin + cyclophosphamide)	[155]
ICIs (Anti PD-1)	NCT03036488	II	pCR: 64.8% (95% CI, 59.9%- 69.5%) incidence of treatment-related adverse events of grade 3 or higher: 78.0%	pCR:51.2%(95%CI,44.1%-58.3%) incidence of treatment-related adverse events of grade 3 or higher: 73.0%	Pembrolizumab + Chemotherapy (paclitaxel + carboplatin)	Placebo + Chemotherapy (paclitaxel + carboplatin)	[156]
ICIs (Anti PD-1)	NCT02622074	I	pCR: 60% (95% CI 49%-71%)		Pembrolizumab + Chemotherapy		[157]
ICIs (Anti PD-1) AXL inhibitor	NCT03184558	I	PFS: 13.1 months (95% CI, 12.4–18.3) OS: 32.0 months (95% CI,13.6–37.1) DCR: 3.4%		Pembrolizumab + Bemcentinib		
ICIs (Anti PD-1) IDO1 inhibitor	NCT02178722	I/II	ORR: 11.1%		MK-3475 + INCB024360		
ICIs (Anti PD-1) IDO1 inhibitor GITR inhibitor	NCT03277352	I/II	ORR: 30% DCR: 70% OS: 25.59 months		Pembrolizumab + Epacadostat + INCAGN01876		
ICIs (Anti PD-1)	NCT01848834	I	ORR: 18.5%		Pembrolizumab		[158]
ICIs (Anti PD-L1)	NCT01375842	I	ORR in first-line: 24% ORR in second-line: 6%		Atezolizumab		[159]
ICIs (Anti PD-1)	NCT02838823	I	ORR: 5% mPFS: 1.8 months (95% CI, 1.4 -4.6)		Humanized anti-PD-1 monoclonal antibody		[160]
ICIs (Anti PD-L1)	NCT02447003 (Group A)	II	mPFS: 2.0 months (95% CI,1.9–2.0) mOS: 9.0 months (95% CI, 7.6–11.2)		Pembrolizumab		[161]
ICIs (Anti PD-L1)	NCT02447003 (Group B)	II	ORR: 21.4% (95% CI 13.9–31.4)		Pembrolizumab		[162]
ICIs (Anti PD-1)	NCT02499367	II	ORR: 20.0%; mPFS: 1.9 months		Nivolumab		[163]
ICIs (Anti PD-L1)	NCT02555657	III	mOS: 12.7 months (95% CI, 9.9–16.3)	mOS: 11.6 months (95% CI, 8.3–13.7)	Pembrolizumab	Chemotherapy	[164]

Clinicaltrials.gov, accessed on November 1, 2021

## Conclusion and perspective

TNBC is still the refractory subtype of breast cancer with the worst prognosis due to the resistance and insensitivity to radiotherapy and chemotherapy. Now, surgery combined with radiotherapy and chemotherapy is the main treatment for patients with TNBC. Good news is that several dysfunctional signaling pathways and proteins have been observed in patients with TNBC, such as PI3K/AKT/mTOR, MAPK signaling pathways, CDKs 4/6, notching signaling, PARP, EMT-associated pathways and AR, which can be used as novel therapeutic targets and some specific agents have received FDA approval. And with improved knowledge of immune checkpoints, effective immunotherapy has gradually applied in TNBC patients. At present, there are amounts of ongoing trials testing the efficacy of targeted agents and ICIs for patients with TNBC. It’s a long and tough way for clinicians to make effective and safe drugs into clinics.

Patients with TNBC show different clinical response to treatment due to the high heterogeneity of TNBC. There are six subtypes of TNBC, and patients with different subtypes have been found to show different responses to drugs. For example, as mentioned above, compares with other subtypes, LAR subtype is more sensitive to CDK4/6 inhibitors and AR antagonist. Immunotherapy is more appropriate for PD-L1 positive patients. For both clinicians and patients, how to predict the clinical efficacy of drugs, and which therapeutic schedule to choose is in need of guidance. Thus, future development of new predictive biomarkers is urgently needed for selecting patients who will benefit most from a particular therapy.

Combination therapy has shown better performance than monotherapy in multiple clinical trials. For example, the concomitant administration of chemotherapy with TGF-β -targeted drugs which have been found to improve the chemotherapy-resistance and recurrence, the combination of AR antagonists with PI3K/mTOR inhibitors, the combination of ICIs and PARPis which can enhance PD-L1 expression in TNBC cells, and other combinations are in clinical trials. However, the mechanisms of combination therapies are not fully understood. More molecular research is necessary to figure out the mechanisms of combination therapy to explore better combination options, including but not limited to the combination among immunotherapy, targeted therapy, and chemoradiotherapy. Furthermore, for combination therapy, the sequence and timing of medications are also worth exploring. Whether different modes of administration will affect the therapeutic effect for the same combination. Postoperative adjuvant, neoadjuvant, or maintenance therapy. Current clinical evidence is insufficient to determine whether postoperative adjuvant, neoadjuvant, or sequential therapy is more satisfactory. And what’s the administration sequence of sequential therapy?

In addition to the development of new drugs, AEs and resistance are also worth studying. For some drugs, AEs limit their application in clinical. This happens a lot when many patients terminate treatment because they can’t tolerate treatment-related side effects. Serious side effects can even lead to death. So, more attention should also be paid to AEs early in trials. After drugs are widely applied to the clinic, patients are prone to resistance in the later course of treatment which is a great challenge for clinical application. Resistance has been observed with several targeted agents in patients with other solid tumors. Except for further figuring out dominant mechanisms of resistance within TNBC, new predictive biomarkers are also necessary to predict resistance. It’s essential to ensure the long-term safety and tolerability of the therapeutic regimens.

In spite of extensive clinical studies on precise medicine in TNBC, whether drugs based on signaling pathways and immune checkpoints can be applied widely in clinical practice or whether they can really improve PFS of the TNBC patients need more data and studies. In the future, we hope to achieve the individualized and precise treatment of TNBC through targeted therapy and immunotherapy.

## Supplementary Information

**Additional file 1: Table 1.** Trials of therapy in triple-negative breast cancer.

## Data Availability

Not applicable. **Code availability** Not applicable.

## Abbreviations

ADC: Antibody–drug conjugate
AE: Adverse event
APC: Adenomatous polyposis coli
AR: Androgen receptor
CDK: Cyclin-dependent kinase
CK1α: Casein kinase 1α
CSC: Cancer stem-like cell
CSF-1: Colony-stimulating factor-1
CTLA-4: T-lymphocyte-associated protein 4
DCP: Disease Control Rate
Dll: Delta-like
Dvl: Dishevelled
FDA: U.S. Food and Drug Administration
FGFR: Fibroblast growth factor receptor
Fzds: Frizzleds
EGFR: Epidermal growth factor receptor
EMT: Epithelial-mesenchymal transition
ER: Estrogen receptor
ERK: Extracellular signal-regulated kinases
GSI: γ-Secretase inhibitors
GSK-3β: Glycogen synthase kinase 3β
HER2: Human epidermal growth factor receptor 2
HR: Hormone receptor
HSP: Heat shock proteins
IGFR: Insulin-like growth factor receptor
JAG: Jagged
LAR: Luminal androgen receptor
LRP5/6: Lipoprotein receptor-related protein 5/6
MAPK: Mitogen-activated protein kinase
mPFS: Median progression free survival
mTOR: Mammalian target of rapamycin
OS: Overall survival
ORR: Objective Response Rate
PARP: Poly-(ADP)-Ribose Polymerase
PARPis: Poly-(ADP)-Ribose Polymerase inhibitors
pCR: Pathological complete response
PD-1: Programmed cell death protein 1
PD-L1: Programmed cell death-ligand 1
PFS: Progression free survival
PI3K: Phosphatidylinositol-3-kinase
PIP2: Phosphorylates 4,5-phosphoinositide
PIP3: Phosphorylates 3,4,5-phosphoinositide
PR: Progesterone receptor
PTEN: Phosphatase and tension homolog
Rb: Retinoblastoma protein
RR: Response rate
RTK: Receptor tyrosine kinase
SG: Sacituzumab govitecan
TEAEs: Treatment-emergent adverse events
TGF-β: Transforming growth factor-beta
TKI: Tyrosine kinase inhibitor
TNBC: Triple negative breast cancer
Trop-2: Trophoblast cell-surface antigen 2
VEGFR: Vascular endothelial growth factor receptor
WIF-1: Wnt inhibitory factor 1
