# Supplementary material for: Therapeutic progress and challenges for triple negative breast cancer: targeted therapy and immunotherapy
Source: Mol Biomed. 2022 Mar 4;3:8. doi: 10.1186/s43556-022-00071-6 (PMC8894518; doi:10.1186/s43556-022-00071-6)
Supplement: Supplementary file 1 — Additional file 1: Table 1. Trials of therapy in triple-negative breast cancer. [file 43556_2022_71_MOESM1_ESM.doc]

**Therapeutic Progress and Challenges for Triple Negative Breast Cancer: Targeted Therapy and Immunotherapy**

Ruoning Yang^1,2, †^, Yueyi Li^1, †^, Hang Wang^1^,, Taolin Qin^3^,, Xiaomeng Yin^1^,, Xuelei Ma^1, *^

1. Department of biotherapy, State Key Laboratory of Biotherapy，Cancer Center, West China Hospital, Sichuan University, , Chengdu, PR China

2. Department of Breast Surgery, Clinical Research Center for Breast, West China Hospital, Sichuan University, Chengdu 610041, China

3. West China Hospital, West China Medical School Sichuan University, Chengdu, PR China

† These authors contributed equally to this work

* **Correspondence to:** Xuelei Ma, West China Hospital, 37 Guoxue Alley, Chengdu 610041, PR China;

Tel: +86-28-85475576; Fax: +86-28-85502796; E-mail: [drmaxuelei@gmail.com](mailto:drmaxuelei@gmail.com)

**Supplementary Table 1: Trials of therapy in triple-negative breast cancer**

| **NCT Number** | **Title** | **Other IDs:** | **Recruitment Status**  **Study Results** | **TNBC Patients Conditions** | **Phase** | **Num. ^*^** | **Study Start Dates**  **(month/day/ year)** | **Therapeutic approach** | **Drugs** |
| --- | --- | --- | --- | --- | --- | --- | --- | --- | --- |
| **NCT04755868** | Talazoparib Maintenance Therapy in Triple- negative Breast Cancer | 4-2020-1131 | Not recruiting  No Results | TNBC | Ⅱ | 206 | March 2021 | PARPi | Talazoparib |
| **NCT02316457** | RNA-Immunotherapy of IVAC_W_bre1_uID and IVAC_M_uID | BN_0002-01 | Active, not recruiting  No Results | TNBC | Ⅰ | 42 | October 2016 | Vaccine | IVAC_W_bre1_uID+VAC_M_uID |
| **NCT02819518** | Study of Pembrolizumab (MK-3475) Plus Chemotherapy vs. Placebo Plus Chemotherapy for Previously Untreated Locally Recurrent Inoperable or Metastatic Triple Negative Breast Cancer (MK-3475-355/KEYNOTE-355) | 3475-355  2016-001432-35  163422  MK-3475-355  KEYNOTE-355 | Active, not recruiting  No Results | TNBC | Ⅲ | 882 | July 27, 2016 | ICIs (Anti PD-1) | Pembrolizumab |
| **NCT03358004** | the Role of Two Different Metronomic Chemotherapy Regimens in Locally Advanced or Metastatic Triple Negative Breast Cancer Patients (TNBC) as Maintenance Therapy After First Line Treatment | IRFMN-BRC-6992 | Terminated  No Results | TNBC | Ⅱ | 4 | June 14, 2017 | Chemotherapy | Vinorelbine Tartrate,  Capecitabine |
| **NCT05064280** | Phase II Study of Pembrolizumab in Combination With Lenvatinib in Patients With TNBC, NSCLC, and Other Tumor Types and Brain Metastases | 2021-0611 | Not recruiting  No Results | TNBC with Brain  Metastases | Ⅱ | 104 | December 31, 2021 | ICI (Anti PD-1)  VEGFR inhibitor | Pembrolizumab, Lenvatinib |
| **NCT04907344** | Study of Camrelizumab Plus Chemotherapy as Neoadjuvant Therapy in Participants With Triple Negative Breast Cancer (TNBC) | MA-BC-II-024 | Not recruiting  No Results | TNBC | Ⅱ/ Ⅲ | 420 | June 15, 2021 | ICIs (Anti PD-1) | Camrelizumab |
| **NCT03109080** | Olaparib & Radiation Therapy for Patients Triple Negative Breast Cancer (TNBC) | IC2016-01 RadioPARP  2016-001837-28 | Active, not recruiting  No Results | TNBC | Ⅰ | 24 | July 24, 2017 | PARPi  adiotherapy | Olaparib + radiation therapy |
| **NCT02499367** | Nivolumab After Induction Treatment in Triple-negative Breast Cancer (TNBC) Patients | N15TON | Active, not recruiting  No Results | TNBC | Ⅱ | 84 | August 2015 | ICIs (Anti PD-1) | Nivolumab |
| **NCT03361800** | Window of Opportunity Trial of Entinostat in Patients With Newly Diagnosed Stage I- IIIC, TNBC | LCCC 1639  P50CA058223 | Terminated  No Results | Stage I-IIIC, TNBC | Ⅰ | 5 | November 28, 2018 | HDAC inhibitor | Entinostat |
| **NCT04405505** | A Study of TQB2450 Injection Combined With Anlotinib Hydrochloride Capsule Versus Paclitaxel for Injection (Albumin Bound) in Subjects With Triple Negative Breast Cancer (TNBC) | TQB2450-III-06 | Not recruiting  No Results | TNBC | Ⅲ | 332 | June 1, 2020 | ICIs (Anti PD-L1)  VEGFR inhibitor | TQB2450+Anlotinib |
| **NCT02368691** | Efficacy and Safety of GTx-024 in Patients With Androgen Receptor-Positive Triple Negative Breast Cancer (AR+ TNBC) | G200901 | Terminated  Has Results | AR+ TNBC | Ⅱ | 32 | June 2015 | SARM | GTx-024  (Enobosarm) |
| **NCT04191135** | Study of Olaparib Plus Pembrolizumab Versus Chemotherapy Plus Pembrolizumab After Induction with First-Line Chemotherapy Plus Pembrolizumab in Triple Negative Breast Cancer (TNBC) (MK-7339-009/KEYLYNK-009) | 7339-009  2019-001892-35  MK-7339-009  KEYLYNK-009  195082 | Active, not recruiting  No Results | TNBC | Ⅱ/ Ⅲ | 1225 | December 19, 2019 | ICIs (Anti PD-1)  PARPI | Pembrolizumab+Olaparib |
| **NCT02456857** | Liposomal Doxorubicin, Bevacizumab, and Everolimus in Patients With Locally Advanced TNBC With Tumors Predicted Insensitive to Standard Chemotherapy; A Moonshot Initiative | 2015-0087  NCI-2015-01556 | Active, not recruiting  No Results | TNBC with tumors predicted insensitive to standard chemotherapy | Ⅱ | 37 | January 12, 2016 | RTK inhibitor+  mTOR inhibitor  chemotherapy | Bevacizumab+ Everolimus +  ATI-0918 |
| **NCT03036488** | Study of Pembrolizumab (MK-3475) Plus Chemotherapy vs Placebo Plus Chemotherapy as Neoadjuvant Therapy and Pembrolizumab vs Placebo as Adjuvant Therapy in Participants With Triple Negative Breast Cancer (TNBC) (MK-3475-522/ KEYNOTE-522) | 3475-522  2016-004740-11  173567  MK-3475-522  KEYNOTE-522 | Active, not recruiting  No Results | TNBC | Ⅲ | 1174 | March 7, 2017 | ICIs (Anti PD-1)  hemotherapy | Pembrolizumab+  Chemotherapy  (Carboplatin+ Paclitaxel+Doxorubicin+Epirubicin+Cyclophosphamide) |
| **NCT04301739** | to Evaluate Efficacy and Safety of HLX10 in Combination With Chemotherapy Versus Placebo in Combination With Chemotherapy as Neoadjuvant Therapy and HLX10 Versus Placebo as Adjuvant Therapy in Patients With Triple Negative Breast Cancer (TNBC) | HLX10-013-TNBCneo | Not recruiting  No Results | TNBC | Ⅲ | 22 | April 17, 2020 | ICIs (Anti PD-1)  Chemotherapy | HLX10+ Chemotherapy(doxorubicin or epirubicin cyclophosphamide) |
| **NCT02393794** | Cisplatin Plus Romidepsin & Nivolumab in Locally Recurrent or Metastatic Triple Negative Breast Cancer (TNBC) | IIT-2014-  CISRomiNivoTNBC  RM-CL-BRST-PI-002783  IIT-2014-PS-BRST-  CISRomiTNBC | Suspended  No Results | Locally Recurrent or Metastatic TNBC | Ⅰ/ Ⅱ | 54 | July 17, 2015 | ICIs (Anti PD-1)  HDAC inhibitors  Chemotherapy | Nivolumab+  Romidepsin+ Cisplatin |
| **NCT02802423** | Phase I/II, Evaluate the Safety and Efficacy of BLEX 404 With Docetaxel in Patients With Advanced/Metastatic Triple Negative Breast Cancer. | BLI-1401-2-01 | Not recruiting  No Results | Advanced/Metastatic TNBC | Ⅰ/ Ⅱ | 44 | January 1, 2022 | Chemotherapy | BLEX 404  +Docetaxel |
| **NCT04230109** | Sacituzumab Govitecan In TNBC | 19-578 | Active, not recruiting  No Results | Localized TNBC | Ⅱ | 51 | July 7, 2020 | ADC  ICIs (Anti PD-1) | SG+ Pembrolizumab |
| **NCT03090165** | Ribociclib and Bicalutamide in AR+ TNBC | BTCRC BRE15-024 | Active, not recruiting  No Results | AR+ TNBC | Ⅰ/ Ⅱ | 37 | March 2, 2017 | CDK inhibitor+ AR antagonist | Ribociclib+ Bicalutamide |
| **NCT04809779** | PD-1 Inhibitor Concurrent With Chemotherapy as Neoadjuvant Therapy for TNBC | 2020466 | Not recruiting  No Results | TNBC | Ⅱ | 49 | March 2021 | ICIs (Anti PD-1) | Sintilimab |
| **NCT03318562** | A PD Study of Oral eFT508 in Subjects With Advanced TNBC and HCC | eFT508-0008 | Terminated  No Results | Metastatic or locally advanced and  unresectable TNBC | Ⅱ | 3 | November 21, 2017 | MNK inhibitor | eFT508 |
| **NCT04517292** | Eribulin Plus Cisplatin Versus Gemcitabine Plus Cisplatin in Triple Negative Breast Cancer (TNBC) | GEP | Not recruiting  No Results | TNBC | Ⅱ | 160 | October 8, 2020 | Chemotherapy | (Eribulin+cisplatin) vs.  (Gemcitabine+  cisplatin) |
| **NCT04303741** | A Phase II Trial of Camrelizumab in Combination With Apatinib and Eribulin in Patients With Advanced TNBC | Immuno2020-01 | Active, not recruiting  No Results | Advanced TNBC | Ⅱ | 46 | March 25, 2020 | ICIs (Anti PD-1)  TKIs (VEGF)  Chemotherapy | Camrelizumab+  Apatinib+  Eribulin |
| **NCT05097248** | Camrelizumab in Combination With PLD and Losartan in Patients With TNBC Who Have Received # 1 Line of Chemotherapy | WHUH-BC-001 | Not recruiting  No Results | TNBC | Ⅱ | 52 | October 2021 | ICIs (Anti PD-1) | Camrelizumab+  Liposomal  doxorubicin+  Losartan |
| **NCT02546934** | ABX Plus Cisplatin Versus Gemcitabine Plus Cisplatin in Triple Negative Breast Cancer (TNBC) | Fudan BR2015-18  CBCSG018 | Active, not recruiting  No Results | TNBC | Ⅲ | 254 | March 2016 | Chemotherapy | ABX + Cisplatin. |
| **NCT03777579** | A Study of First-line JS001 and Nab- paclitaxel Versus Palcelbo and Nab- Paclitaxel in Participants With Advanced Recurrent or Metastatic TNBC | NABP201801 | Suspended  No Results | Advanced Recurrent or Metastatic TNBC | Ⅲ | 375 | December 21, 2018 | ICIs (Anti PD-1) | JS001+  Nab-Paclitaxel, |
| **NCT01234532** | Entinostat and Anastrozole in Treating Postmenopausal Women With TNBC That Can Be Removed by Surgery | HP-00047658  NCI-2011-02542  CDR0000687507  8597 | Terminated  No Results | Postmenopausal  Women With TNBC  That Can Be Removed  by Surgery | Ⅱ | 5 | October 2010 | HDAC inhibitor+  aromatase inhibitor | Entinostat+ anastrozole |
| **NCT03125902** | A Study of Atezolizumab and Paclitaxel Versus Placebo and Paclitaxel in Participants With Previously Untreated Locally Advanced or Metastatic Triple Negative Breast Cancer (TNBC) | MO39196  2016-004024-29 | Active, not recruiting  Has Results | Previously Untreated Locally Advanced or Metastatic TNBC | Ⅲ | 651 | August 25, 2017 | ICIs (Anti PD-1) | Atezolizumab+ Paclitaxel |
| **NCT03487666** | OXEL: Immune Checkpoint or Capecitabine or Combination Therapy as Adjuvant Therapy for TNBC With Residual Disease | 2017-1535 | Active, not recruiting  No Results | TNBC | Ⅱ | 45 | May 21, 2018 | ICIs (Anti PD-1),  Chemotherapy | Nivolumab+ Capecitabine |
| **NCT04249167** | Cryoablation, Atezolizumab/Nab-paclitaxel for Locally Advanced or Metastatic Triple Negative Breast Cancer | MC2031  NCI-2020-00333  19-008704  P30CA015083 | Active, not recruiting  No Results | Locally Advanced or  Metastatic TNBC | Ⅰ | 5 | January 23, 2020 | ICIs (Anti PD-1)  Surgery | Cryoablation+ atezolizumab+ nab-paclitaxel |
| **NCT02315196** | Pegylated Liposomal Doxorubicin Hydrochloride and Carboplatin Followed by Surgery and Paclitaxel in Treating Patients With Triple Negative Stage II-III Breast Cancer | Pro20140000477  NCI-2014-02029  041401  P30CA072720 | Suspended  No Results | Stage II-III TNBC | Ⅱ | 60 | December 26, 2014 | Chemotherapy  Surgery | Doxil+ carboplatin+ surgery + paclitaxel |
| **NCT02464774** | Breast-Conserving Therapy in Patients With Triple-Negative Breast Cancer | 2014-FXY-093 | Not recruiting  No Results | TNBC | ---- | 464 | July 2015 | Surgery | Breast-Conserving Therapy |
| **NCT02883062** | Carboplatin and Paclitaxel With or Without Atezolizumab Before Surgery in Treating Patients With Newly Diagnosed, Stage II-III Triple-Negative Breast Cancer | NCI-2016-01301  201706104  10013  UM1CA186689  UM1CA186704 | Active, not recruiting  No Results | Stage II-III TNBC | Ⅱ | 72 | May 17, 2017 | Chemotherapy  ICIs (Anti PD-L1) | Atezolizumab+  Chemotherapy (carboplatin) |
| **NCT04734262** | A Phase II Study to Explore the Safety, Tolerability, and Preliminary Antitumor Activity of Sitravatinib and Tislelizumab in Patients With Locally Recurrent or Metastatic Triple Negative Breast Cancer | BGB-900-2001-IIT | Not recruiting  No Results | Locally Recurrent or Metastatic TNBC | Ⅱ | 61 | January 30, 2021 | RTK inhibitor+  CIs (Anti PD-1) | Sitravatinib+ Tislelizumab |
| **NCT03362060** | PVX-410 Vaccine Plus Pembrolizumab in HLA-A2+ Metastatic Triple Negative Breast Cancer | 17-328 | Active, not recruiting  No Results | Metastatic TNBC | Ⅰ | 20 | December 12, 2017 | ICIs (Anti PD-1)  Vaccine | Pembrolizumab+ PVX-410 |
| **NCT04634747** | Phase 2 Study to Evaluate PVX-410 + Pembrolizumab + Chemotherapy for Metastatic, PD-L1+ Triple-Negative Breast Cancer | 2020-001 | Not recruiting  No Results | Metastatic, PD-L1+ TNBC | Ⅱ | 53 | November 1, 2021 | ICIs (Anti PD-1)  Vaccine  Chemotherapy | PVX-410+  pembrolizumab+ chemotherapy |
| **NCT02971761** | Pembrolizumab and Enobosarm in Treating Patients With Androgen Receptor Positive Metastatic Triple Negative Breast Cancer | 16131  NCI-2016-01759 | Active, not recruiting  No Results | AR+ Metastatic TNBC | Ⅱ | 29 | June 1, 2017 | SARM+  ICIs (Anti PD-1) | Enobosarm +Pembrolizumab |
| **NCT01930292** | Debio 1143 in Combination With Carboplatin and Paclitaxel in Patient With Advanced Solid Malignancies | Debio 1143-103  2012-003676-40 | Terminated  No Results | Basal-like/Claudin Low TNBC | Ⅰ | 31 | April 2013 | IAPs  Chemotherapy | Debio 1143  +Chemotherapy (Paclitaxel+Carboplatin) |
| **NCT03199040** | Neoantigen DNA Vaccine Alone vs. Neoantigen DNA Vaccine Plus Durvalumab in Triple Negative Breast Cancer Patients Following Standard of Care Therapy | 201710109  1R01CA240983-01 | Active, not recruiting  No Results | TNBC | Ⅰ | 10 | August 8, 2019 | Vaccine  ICIs (Anti PD-L1) | Neoantigen DNA vaccine+  Durvalumab |
| **NCT02698176** | A Dose Exploration Study With Birabresib (MK-8628) in Participants With Selected Advanced Solid Tumors (MK-8628-006) | 8628-006  MK-8628-006  2015-005488-18 | Terminated  Has Results | Advanced TNBC | Ⅰ | 13 | May 4, 2016 | inhibitor of BET Proteins | Birabresib |
| **NCT03330405** | Javelin Parp Medley: Avelumab Plus Talazoparib In Lcally Advanced Or Metastatic Solid Tumors | B9991025 2017-001509-33 JAVELIN PARP MEDLEY | Active, not recruiting  No Results | Locally Advanced or Metastatic TNBC | Ⅱ | 226 | October 19, 2017 | ICIs (Anti PD-L1)  ARPi | Avelumab+ Talazoparib |
| **NCT03281954** | Clinical Trial of Neoadjuvant Chemotherapy With Atezolizumab or Placebo in Patients With Triple-Negative Breast Cancer Followed After Surgery by Atezolizumab or Placebo | NSABP B-59/GBG 96-  GeparDouze  2017-002771-25  MO39875 | Active, not recruiting  No Results | TNBC | Ⅲ | 1520 | December 19, 2017 | ICIs (Anti PD-L1) | Atezolizumab |
| **NCT03875313** | Study of CB-839 (Telaglenastat) in Combination With Talazoparib in Patients With Solid Tumors | CX-839-011 | Terminated  No Results | TNBC | Ⅰ/ Ⅱ | 33 | May 20, 2019 | Glutaminase inhibitor  PARPi | CB-839+  Talazoparib |
| **NCT04424641** | A Study on the Safety of GEN1044 (DuoBody®-CD3x5T4) in Subjects With Malignant Solid Tumors | GCT1044-01 | Active, not recruiting  No Results | Locally Advanced or Metastatic TNBC | Ⅰ/ Ⅱ | 37 | July 15, 2020 | IgG1 bispecific anti CD3 and 5T4 | GEN1044 |
| **NCT02547987** | Neoadjuvant Carboplatin and Docetaxel in Triple Negative Breast Cancer | H- 36960 CADENCE | Active, not recruiting  Has Results | TNBC | Ⅱ | 25 | November 1, 2015 | Chemotherapy | Docetaxel + Carboplatin |
| **NCT02676986** | Short-term Preoperative Treatment With Enzalutamide, Alone or in Combination With Exemestane in Primary Breast Cancer | 009684QM  2014-002001-37 | Active, not recruiting  No Results | AR+ TNBC | Ⅱ | 221 | August 2015 | AR antagonist | Enzalutamide ±Exemestane |
| **NCT02833766** | Anti-EGFR-immunoliposomes Loaded With Doxorubicin in Patients With Advance Triple Negative EGFR Positive Breast Cancer | SAKK 24/14 | Terminated  No Results | EGFR+ TNBC | Ⅱ | 48 | October 28, 2016 | anti-EGFR | anti-EGFR-IL-dox |
| **NCT02779855** | Talimogene Laherparepvec in Combination With Neoadjuvant Chemotherapy in Triple Negative Breast Cancer | MCC-18621 | Active, not recruiting  Has Results | TNBC | Ⅰ/ Ⅱ | 50 | May 2, 2017 | Chemotherapy | Talimogene laherparepvec+ Chemotherapy (Paclitaxel) |
| **NCT04739670** | Evaluating the Efficacy and Safety of Bevacizumab, Carboplatin, Gemcitabine and Atezolizumab in Breast Cancer | 19/002 | Not recruiting  No Results | Metastatic TNBC | Ⅱ | 31 | February 2021 | ICIs (Anti PD-L1)  Anti-VEGFR  Chemoptherapy | Atezolizumab+ Bevacizumab+ Gemcitabine+ Carboplatin |
| **NCT03901469** | A Study of ZEN003694 and Talazoparib in Patients With Triple Negative Breast Cancer | ZEN003694-004  2018-003906-26 | Active, not recruiting  No Results | TNBC | Ⅱ | 49 | June 26, 2019 | Bromodomain inhibitor  PARPi | ZEN003694+ Talazoparib |
| **NCT02890069** | A Study of PDR001 in Combination With LCL161, Everolimus or Panobinostat | CPDR001X2102 | Active, not recruiting  No Results | TNBC | Ⅰ | 298 | October 14, 2016 | ICIs (Anti PD-1)  IAP inhibitor  mTOR inhibitor  HDAC inhibitor | PDR001+LCL161/Everolimus+ Panobinostat |
| **NCT02078752** | A Study Of PF-06647263 In Patients With Advanced Solid Tumors | B7521001 | Terminated  Has Results | Advanced TNBC | Ⅰ | 60 | April 9, 2014 | ADC (anti-EFNA4) | PF-06647263 |
| **NCT01745367** | Tivozanib in Combination With Paclitaxel in Patients With Locally Recurrent or Metastatic Triple Negative Breast Cancer | AV-951-12-204  2012-003507-35 | Terminated  Has Results | TNBC | Ⅱ | 30 | November 2012 | VEGF inhibitor | Paclitaxel±  Tivozanib |
| **NCT02158507** | Pilot Study of Veliparib (ABT-888) and Lapatinib (Tykerb) in Patients With Metastatic, Triple Negative Breast Cancer | F131219003(UAB 1372)  000504723 | Active, not recruiting  No Results | Metastatic TNBC | -- | 23 | September 2014 | PAPRi  EGFR inhibito | Veliparib+ Lapatinib |
| **NCT03071757** | A Study of the Safety, Tolerability and Pharmacokinetics of ABBV-368 as a Single Agent and Combination in Subjects With Locally Advanced or Metastatic Solid Tumors | M16-074  2016-004205-14 | Active, not recruiting  No Results | Advanced TNBC | Ⅰ | 170 | March 21, 2017 | anti-OX40  ICIs (anti PD-1) | ABBV-368+ ABBV-181 |
| **NCT02554812** | A Study of Avelumab In Combination With Other Cancer Immunotherapies In Advanced Malignancies (JAVELIN Medley) | B9991004  2015-002552-27  JAVELIN MEDLEY | Active, not recruiting  No Results | Advanced Cancer | Ⅱ | 398 | November 9, 2015 | ICIs（PD-L1）  Anti-4-1BB antibody | Avelumab+ Utomilumab |
| **NCT04395989** | An Umbrella Trial Based on Molecular Pathway for Patients With Metastatic TNBC. | SCHBCC-N031 | Recruiting  No Results | TNBC | Ⅱ | 138 | May 15, 2020 | Umbrella Trials | A1:Pyrotinib+Capecitabine  A2: nab-paclitaxel  B1:everolimus+nabpaclitaxel  B2: nab-paclitaxel  C1:PD-1+nab-paclitaxel+famitinib  C2: nab-paclitaxel  D1:VEGFR+nab-paclitaxel  D2: nab-paclitaxel  DE1: Everolimus + nab-paclitaxel  E2: nab-paclitaxel |
| **NCT03742349** | Study of Safety and Efficacy of Novel Immunotherapy Combinations in Patients With Triple Negative Breast Cancer (TNBC). | CADPT01A12101C  2018-002244-82 | Recruiting  No Results | TNBC | Ⅰ | 220 | January 31, 2019 | ICIs (anti PD-1) | Spartalizumab+LAG525+NIR178/capmatinib/ MCS110/canakinumab |
| **NCT05088057** | Neoadjuvant Camrelizumab Plus Chemotherapy in Triple Negative Breast Cancer | 2021-TNBC-01 | Recruiting  No Results | TNBC | Ⅱ | 30 | September 20, 2021 | ICIs (anti PD-1)  Chemotherapy | Camrelizumab+Chemotherapy  (Doxorubicin Cyclophosphamide+ Docetaxel) |
| **NCT04799249** | Trilaciclib, a CDK 4/6 Inhibitor, in Patients Receiving Gemcitabine and Carboplatin for Metastatic Triple-Negative Breast Cancer (TNBC) | G1T28-208  2020-004930-39 | Recruiting  No Results | Metastatic TNBC | Ⅲ | 250 | April 15, 2021 | Chemotherapy  CDK inhibitor | Chemotherapy (gemcitabine+carboplatin)+ Trilaciclib |
| **NCT03911973** | A Study to Evaluate the Efficacy and Safety of SG001 in Combination With Nab- Paclitaxel in Patients With Advanced Triple- Negative Breast Cancer (TNBC) | BTCRC-BRE18-337 | Recruiting  No Results | TNBC | Ⅰ/ Ⅱ | 54 | April 17, 2019 | PARPi  PI3K inhibitor | Talazoparib+ Gedatolisib |
| **NCT05068141** | A Study to Evaluate the Efficacy and Safety of SG001 in Combination With Nab- Paclitaxel in Patients With Advanced Triple- Negative Breast Cancer (TNBC) | SYSA1802-CSP-006 | Recruiting  No Results | TNBC | Ⅱ | 79 | October 1, 2021 | ICIs (Anti PD-1) | SG001+ Nab-paclitaxel |
| **NCT03752723** | Study of GX-I7 in Combination With Pembrolizumab in Refractory or Relapsed (R/R) NBC Subjects (GX-I7-CA-006/ KEYNOTE-899) | GX-I7-CA-006 | Recruiting  No Results | TNBC | Ⅰ/ Ⅱ | 83 | March 27, 2019 | Human IL-7 and hyFc  ICIs (Anti PD-1)  Chemotherapy | (GX-I7+Pembrolizumab) +cyclophosphamide |
| **NCT03805399** | FUSCC Refractory TNBC Umbrella (FUTURE) | 1807188-16 | Recruiting  No Results | TNBC | Ⅰ/ Ⅱ | 140 | July 17, 2018 | Umbrella Trials | A: Pyrotinib+Capecitabine  B: ARinhibitor+CDK4/6 inhibitor  C: anti-PD-1+nab-paclitaxel  D: PARPi  E: BLIS  +anti-VEGFR  F: MES  +anti-VEGFR  F: mTOR inhibitor  +nab-paclitaxel |
| **NCT04468061** | Sacituzumab Govitecan +/- Pembrolizumab in Metastatic TNBC | 20-166 | Recruiting  No Results | Metastatic TNBC | Ⅱ | 110 | July 20, 2020 | ADC (Anti Trop-2)  ICIs (anti PD-1) | SG+  Pembrolizumab |
| **NCT03567720** | Tavo and Pembrolizumab With or Without Chemotherapy in Patients With Inoperable Locally Advanced or Metastatic TNBC | OMS-I141  (KEYNOTE-890)  KEYNOTE-890  MK3475-890 | Recruiting  No Results | TNBC | Ⅱ | 65 | October 11, 2018 | ICIs (Anti PD-1)  Chemotherapy | Tavo-EP+ pembrolizumab +chemotherapy |
| **NCT04676997** | Neoadjuvant Study of Camrelizumab Plus Chemotherapy in Triple Negative Breast Cancer (TNBC) | MA-BC-II-006 | Recruiting  No Results | TNBC | Ⅱ | 20 | May 20, 2020 | ICIs (Anti PD-1)  Chemotherapy | Camrelizumab+  Chemotherapy (Nabpaclitaxel+Epirubicin+Cyclophosphamide) |
| **NCT05103917** | A Study to Assess the Safety, Tolerability and Antitumor Activity of X4P-001 in Combination With TNBC | X4P-001-201 | Enrolling by invitation  No Result | TNBC | Ⅰ/ Ⅱ | 24 | July 21, 2021 | CXCR4 inhibitor | X4P-001 |
| **NCT04159818** | Immune Induction Strategies to Improve Response to Immune Checkpoint Blockade in Triple Negative Breast Cancer (TNBC) Patients | N19TON | Recruiting  No Results | Metastatic TNBC | Ⅱ | 52 | February 21, 2020 | ICIs (Anti PD-1) | Nivolumab+  Cisplatin/  doxorubicin |
| **NCT03997123** | Capivasertib+Paclitaxel as First Line Treatment for Patients with Locally Advanced or Metastatic TNBC | D3614C00001 | Recruiting  No Results | TNBC | Ⅲ | 924 | June 25, 2019 | Chemotherapy  AKT inhibitor | Paclitaxel+  Capivasertib |
| **NCT04613674** | A Study of Camrelizumab Plus Chemotherapy vs Placebo Plus Chemotherapy as Neoadjuvant Therapy in Participants with Triple Negative Breast Cancer (TNBC) | SHR1210-III-322 | Recruiting  No Results | TNBC | Ⅲ | 581 | December 9, 2020 | Chemotherapy  ICIs (Anti PD-1) | Chemotherapy+  Camrelizumab |
| **NCT02593175** | Women's MoonShot: Neoadjuvant Treatment With PaCT for Patients With Locally Advanced TNBC | 2015-0294  NCI-2015-02183 | Recruiting  No Results | Locally Advanced TNBC | Ⅱ | 37 | August 26, 2016 | Anti EGFR  Chemotherapy | Panitumumab+  paclitaxel+  carboplatin |
| **NCT04427293** | Preoperative Lenvatinib Plus Pembrolizumab in Early-Stage Triple- Negative Breast Cancer (TNBC) | 2020-0232 | Recruiting  No Results | TNBC | Ⅰ | 12 | July 9, 2020 | VEGFR inhibitor  ICIs (Anti PD-1) | Lenvatinib+ Pembrolizumab |
| **NCT03150576** | Platinum and Polyadenine 5'Diphosphoribose Polymerisation (PARP) Inhibitor for Neoadjuvant Treatment of Triple Negative Breast Cancer (TNBC) and/or Germline BRCA (gBRCA) Positive Breast Cancer | A093777 | Recruiting  No Results | TNBC | Ⅱ/Ⅲ | 527 | May 2016 | Chemotherapy  PARPi | Chemotherapy (Paclitaxel+  Carboplatin) + Olaparib |
| **NCT04297267** | A Study to Evaluate Gemcitabine Plus Cisplatin in the Treatment of TNBC Patients Following Neoadjuvant Chemotherapy | 1608162-19-1805B | Recruiting  No Results | TNBC | Ⅱ | 150 | February 7, 2017 | Chemotherapy | Gemcitabine+ Cisplatin |
| **NCT04706962** | TH1902 in Patients With Advanced Solid Tumors | TH1902-CTR-0001 | Recruiting  No Results | TNBC | Ⅰ | 65 | March 4, 2021 | ADC | TH1902 |
| **NCT03562637** | Study of Adagloxad Simolenin (OBI-822)/ OBI-821 in the Adjuvant Treatment of Patients With Globo H Positive TNBC | OBI-822-011 | Recruiting  No Results | TNBC | Ⅲ | 668 | December 5, 2018 | Vaccine | Aloxad simolenin (OBI 822) +  BI-821 |
| **NCT04877821** | The Efficacy and Safety of Sintilimab Plus Anlotinib Combined With Chemotherapy as Neoadjuvant Therapy in TNBC | 2021-19 | Recruiting  No Results | TNBC | Ⅱ | 46 | September 15, 2021 | ICIs (Anti PD-1)  VEGFR inhibitor  Chemotherapy | Sintilimab+  Anlotinib+  Chemotherapy (Nab-paclitaxel+Carboplatin+Epirubicin+Cyclophosphamide) |
| **NCT04683679** | A Study of Radiation Therapy With Pembrolizumab and Olaparib in Women Who Have Triple-Negative Breast Cancer | 20-505 | Recruiting  No Results | TNBC | Ⅱ | 56 | April 21, 2021 | ICIs (Anti PD-1)  Radiotherapy  PARPi | Pembrolizumab+ Radiation +Olaparib |
| **NCT04674306** | Adjuvant Therapy With an Alpha-lactalbumin Vaccine in Triple-Negative Breast Cancer | CASE6119  W81XWH-17-1-0593  W81XWH-17-1-0592 | Recruiting  No Results | Stage IIA-IIIC TNBC | Ⅰ | 24 | October 1, 2021 | Vaccine | α-lactalbumin |
| **NCT04915755** | Efficacy and Safety Comparison of Niraparib to Placebo in Participants With Either Human Epidermal Growth Factor 2 Negative (HER2-) Breast Cancer Susceptibility Gene Mutation (BRCAmut) or Triple-Negative reast Cancer (TNBC) With Molecular Disease | 213831 | Recruiting  No Results | Tumor BRCA wild type TNBC | Ⅲ | 800 | June 28, 2021 | PARPi | Niraparib |
| **NCT03674827** | Vaccine-Based Immunotherapy Regimen for NSCLC and TNBC | C3621001  VBIR-2 | Recruiting  No Results | TNBC | Ⅰ | 36 | November 27, 2018 | VBIR-2 | PF-06936308 |
| **NCT04335669** | NordicTrip, a Translational Study of eoperative Chemotherapy in TNBC | NBG-19-01  SWEBCG 19-01 | Recruiting  No Results | TNBC | Ⅲ | 820 | December 20, 2019 | Chemotherapy | Cclophosphamide/epirubicin/capecitabine/carboplatin/ paclitaxel |
| **NCT03295552** | Decitabine Plus Carboplatin in theTreatment of Metastatic TNBC | RJBC1701 | Recruiting  No Results | Metastatic TNBC | Ⅱ | 59 | November 15, 2017 | Chemotherapy | Decitabine+ Carboplatin |
| **NCT04434040** | Atezolizumab + Sacituzumab Govitecan to Prevent Recurrence in TNBC (ASPRIA) | 20-028 | Recruiting  No Results | TNBC | Ⅱ | 40 | July 2, 2020 | ICIs (Anti PD-L1)  ADC (anti-Trop-2) | Atezolizumab + SG |
| **NCT04664829** | The Role of Bexarotene in Inducing Susceptibility to Chemotherapy in Metastatic TNBC | BEXMET | Recruiting  No Results | Metastatic TNBC | Ⅰ | 12 | October 1, 2020 | Retinoid Receptor agonist  Chemotherapy | Bexarotene +Capecitabine |
| **NCT04501523** | A Prospective, Phase II Trial Using ctDNA to Initiate Post-operation Boost Therapy After NAC in TNBC | SunYatsenU2H-LQ3 | Recruiting  No Results | TNBC | Ⅱ | 460 | August 3, 2020 | ICIs (Anti PD-1) | Tislelizumab+ capecitabine |
| **NCT04807192** | CMP-001 and Pre-operative Stereotactic Body Radiation Therapy (SBRT) in Early-Stage Triple Negative Breast Cancer (TNBC) | CHUV-DO-0009-  CyberImmunoBreast | Recruiting  No Results | TNBC | Ⅱ | 40 | April 8, 2021 | Radiotherapy  TLR9 agonist | SBRT + CMP-001 |
| **NCT03674242** | Study of Eryaspase in Combination With Chemotherapy Versus Chemotherapy Alone for the Treatment of TNBC (TRYbeCA-2) | GRASPA-TNBC-2018-02 | Recruiting  No Results | TNBC | Ⅱ/Ⅲ | 64 | June 13, 2019 | Chemotherapy  Eryaspase | Chemotherapy (Gemcitabine+Carboplatin) + eryaspase |
| **NCT03876886** | The Trial Comparing Dose-dense AC-T With TP as Adjuvant Therapy for TNBC With Homologous Recombination Repair Deficiency | BXu-1839 | Recruiting  No Results | TNBC | Ⅲ | 200 | February 22, 2019 | Chemotherapy | Epirubicin/ Cyclophosphamide/ Paclitaxel/ Carboplatin |
| **NCT03504488** | CAB-ROR2-ADC Safety and Efficacy Study in Patients With TNBC or Head & Neck Cancer (Ph1) and NSCLC or Melanoma (Ph2) | BA3021-001 | Recruiting  No Results | TNBC | Ⅰ/Ⅱ | 420 | June 27, 2018 | ADC (anti-ROR2) | CAB-ROR2-ADC± |
| **NCT03616886** | Paclitaxel + Carboplatin + Durvalumab With or Without Oleclumab for Previously Untreated Locally Recurrent Inoperable or Metastatic TNBC | IJB-SYNERGY-012017 | Recruiting  No Results | TNBC | Ⅰ/Ⅱ | 171 | December 28, 2018 | Chemotherapy  ICIs (Anti PD-1)  CD73 inhibitor | Chemotherapy(paclitaxel+carboplatin) + durvalumab+oleclumab |
| **NCT04251533** | Study Assessing the Efficacy and Safety of Alpelisib + Nab-paclitaxel in Subjects With Advanced TNBC Who Carry Either a PIK3CA Mutation or Have PTEN Loss | CBYL719H12301  2019-002637-11 | Recruiting  No Results | TNBC | Ⅲ | 566 | June 8, 2020 | PI3K inhibitor | nab-paclitaxel±alpelisib |
| **NCT04085276** | Toripalimab in Combination With Nab-Paclitaxel For Patients With Metastatic or Recurrent Triple-Negative Breast Cancer (TNBC) With or Without Systemic Treatment | JS001-026-III-TNBC | Recruiting  No Results | TNBC | Ⅲ | 660 | December 21, 2018 | ICIs (Anti PD-1) | Nab-Paclitaxel± JS001 |
| **NCT03579472** | M7824 and Eribulin Mesylate in Treating Patients With Metastatic Triple Negative Breast Cancer | 2017-0500  NCI-2018-01140  P30CA016672 | Recruiting  No Results | Metastatic TNBC | Ⅰ | 20 | May 30, 2018 | TGF-β inhibitor  ICIs (Anti PD-L1) | Bintrafusp alfa+ eribulin mesylate |
| **NCT04176848** | CFI-400945 and Durvalumab in Patients With Advanced Triple Negative Breast Cancer | I239 | Recruiting  No Results | Advanced TNBC | Ⅱ | 28 | December 19, 2019 | PLK inhibitor  ICIs (Anti PD-1) | CFI-400945+ Durvalumab |
| **NCT04373031** | Pembrolizumab, IRX-2, and Chemotherapy in Triple Negative Breast Cancer | 2019000486 | Recruiting  No Results | TNBC | Ⅱ | 30 | December 30, 2020 | ICIs (Anti PD-1)  IRX 2  Chemotherapy | Pembrolizumab+ IRX 2  + Chemotherapy |
| **NCT03971409** | Avelumab With Binimetinib, Sacituzumab Govitecan, or Liposomal Doxorubicin in Treating Patients With Stage IV or Unresectable, Recurrent Triple Negative Breast Cancer | 187519  NCI-2019-01531  TBCRC 047  BRE16-279 | Recruiting  No Results | Stage IV or Unresectable, Recurrent TNBC | Ⅱ | 150 | July 8, 2019 | ICIs (Anti PD-1) | Avelumab |
| **NCT03756090** | The Combination of Palbociclib With ddEC-P as Neoadjuvant Therapy in Triple Negative Breast Cancer | PECP | Unknown  No Results | TNBC | -- | 100 | December 1, 2018 | CDK4/6 inhibitor Chemotherapy | Palbociclib+  Chemotherapy (Epirubicin+  Cyclophosphamide+ Paclitaxel) |
| **NCT03130439** | Abemaciclib for Patients With Retinoblastoma-Positive, Triple Negative Metastatic Breast Cancer | 17-024 | Active, not recruiting  No Results | Metastatic TNBC | II | 37 | May 26, 2017 | CDK4/6 inhibitor | Abemaciclib (LY2835219) |
| **NCT02605486** | Palbociclib in Combination with Bicalutamide for the Treatment of AR (+) Metastatic Breast Cancer (MBC) | 15-207 | Active, not recruiting  No Results | AR+ Metastatic TNBC | Ⅰ/Ⅱ | 46 | November 11, 2015 | CDK4/6 inhibitor  AR antagonist | Palbociclib+ Bicalutamide |
| **NCT02978716** | Trilaciclib (G1T28), a CDK 4/6 Inhibitor, in Combination With Gemcitabine and Carboplatin in Metastatic Triple Negative Breast Cancer (mTNBC) | G1T28-04  2016-004466-26 | Active, not recruiting  No Results | Metastatic TNBC | Ⅱ | 102 | February 7, 2017 | CDK4/6 inhibitor  Chemotherapy | Trilaciclib+Chemotherapy（Gemcitabine+ Carboplatin） |
| **NCT01969643** | A Safety Study of SGN-LIV1A in Breast Cancer Patients | SGNLVA-001 | Recruiting  No Results | Metastatic TNBC | Ⅰ | 448 | October 22, 2013 | ADC (Anti LIV-1) | Ladiratuzumab vedotin±Trastuzumab |
| **NCT03310957** | Safety and Efficacy of SGN-LIV1A Plus Pembrolizumab for Patients With Locally-Advanced or Metastatic Triple-Negative Breast Cancer | SGNLVA-002  KEYNOTE 721  2017-002289-35 | Recruiting  No Results | Locally-Advanced of  Metastatic TNBC | Ⅰ/Ⅱ | 161 | February 27, 2018 | ADC (Anti LIV-1)  ICIs (Anti PD-L1) | ladiratuzumab vedotin  + pembrolizumab |
| **NCT04441099** | NBE-002 in Patients With Advanced Solid Tumors | NBE-002-01 | Active, not recruiting  No Results | Advanced TNBC | Ⅰ/Ⅱ | 100 | June 19, 2020 | ADC (Anti ROR1) | NBE-002 |
| **NCT03170960** | Study of Cabozantinib in Combination With Atezolizumab to Subjects With Locally Advanced or Metastatic Solid Tumors | XL184-021 | Recruiting  No Results | Locally Advanced or Metastatic TNBC | Ⅰ/Ⅱ | 1732 | September 5, 2017 | TKIs  ICIs (Anti PD-L1) | Cabozantinib+Atezolizumab |
| **NCT03800836** | A Study to Evaluate the Safety and Efficacy of Ipatasertib in Combination With Atezolizumab and Paclitaxel or Nab-Paclitaxel in Participants With Locally Advanced or Metastatic Triple-Negative Breast Cancer | CO40151  2017-001957-15 | Active, not recruiting  No Results | Locally Advanced or  Metastatic TNBC | Ⅰ | 140 | February 13, 2018 | AKT inhibitor  ICIs (Anti PD-L1)  Chemotherapy | Ipatasertib+Atezolizumab +  Chemotherapy  (Paclitaxel / Nab-Paclitaxel) |
| **NCT04177108** | A Study Of Ipatasertib in Combination With Atezolizumab and Paclitaxel as a Treatment for Participants With Locally Advanced or Metastatic Triple-Negative Breast Cancer. | CO41101  2019-000810-12 | Active, not recruiting  No Results | Locally advanced or metastatic TNBC | Ⅲ | 242 | November 25, 2019 | AKT inhibitor  ICIs (Anti PD-L1)  Chemotherapy | Ipatasertib+ Atezolizumab+  Chemotherapy  (Paclitaxel) |
| **NCT02484404** | Phase I/II Study of the Anti-Programmed Death Ligand-1 Durvalumab Antibody (MEDI4736) in Combination With Olaparib and/or Cediranib for Advanced Solid Tumors and Advanced or Recurrent Ovarian, Triple Negative Breast, Lung, Prostate and Colorectal Can... | 150145  15-C-0145 | Recruiting  No Results | Advanced or Recurrent TNBC | I/II | 384 | June 29, 2015 | PARPi  ICIs (Anti PD-L1) | Durvalumab +olaparib ±cediranib |
| **NCT03544125** | Olaparib and Durvalumab in Treating Participants With Metastatic Triple Negative Breast Cancer | STUDY00018239  NCI-2018-00819 | Completed  No Results | Metastatic TNBC | Ⅰ | 3 | May 3, 2018 | PARPi  ICIs (Anti PD-L1) | Olaparib +Durvalumab |
| **NCT04360941** | PAveMenT: Palbociclib and Avelumab in Metastatic AR+ Triple Negative Breast Cancer (PAveMenT) | CCR4884 | Recruiting  No Results | Metastatic AR+ TNBC | Ⅰ | 255 | August 11, 2020 | CDK inhibitor  ICIs (Anti PD-1) | Palbociclib +Avelumab |
| **NCT02660034** | The Safety, Pharmacokinetics and Antitumor Activity of BGB-A317 in Combination With BGB-290 in Participants With Advanced Solid Tumors | BGB-A317/BGB-290_Study_001  2017-003580-35 | Completed  No Results | Advanced TNBC | Ⅰ | 29 | February 2, 2016 | ICIs (Anti PD-1)  PARPi | Tislelizumab+pamiparib |
| **NCT02826434** | Adjuvant PVX-410 Vaccine and Durvalumab in Stage II/III Triple Negative Breast Cancer | 16-132 | Active, not recruiting  No Results | Stage II/III TNBC | Ⅰ | 22 | August 2016 | Vaccine  ICIs (Anti PD-1) | PVX-410 Vaccine  +Durvalumab |
| **NCT03012100** | Multi-epitope Folate Receptor Alpha Peptide Vaccine, GM-CSF, and Cyclophosphamide in Treating Patients With Triple Negative Breast Cancer | RU011501I  NCI-2016-01878  P30CA015083 | Recruiting  No Results | Stage II/III TNBC | Ⅱ | 280 | March 31, 2017 | Vaccine | Multi-epitope Folate  Receptor Alpha Peptide Vaccine |
| **NCT03606967** | Testing the Addition of an Individualized Vaccine to Nab-Paclitaxel, Durvalumab and Tremelimumab and Chemotherapy in Patients With Metastatic Triple Negative Breast Cancer | NCI-2018-01581  10146  UM1CA186704 | Recruiting  No Results | Metastatic TNBC | Ⅱ | 70 | December 14, 2018 | Vaccine | Personalized Synthetic Long Peptide Vaccine |
| **NCT03371017** | A Study of the Efficacy and Safety of Atezolizumab Plus Chemotherapy for Patients With Early Relapsing Recurrent Triple-Negative Breast Cancer | MO39193  2016-005119-42 | Recruiting  No Results | Early Relapsing Recurrent TNBC | Ⅲ | 572 | January 11, 2018 | ICIs (Anti PD-L1)  Chemotherapy | Atezolizumab+Chemotherapy  (Placebo+Gemcitabine+Capecitabine+Carboplatin) |
| **NCT02954874** | Testing MK-3475 (Pembrolizumab) as Adjuvant Therapy for Triple Receptor-Negative Breast Cancer | NCI-2016-01595  S1418/BR006  s16-02231  S1418  U10CA180888 | Active, not recruiting  No Results | TNBC | Ⅲ | 1155 | November 15, 2016 | ICIs (Anti PD-1)  Radiotherapy | Pembrolizumab+  Radiation Therapy |
| **NCT02926196** | Adjuvant Treatment for High-risk Triple Negative Breast Cancer Patients With the Anti-PD-l1 Antibody Avelumab | A-BRAVE-Trial  2016-000189-45 | Active, not recruiting  No Results | High-risk TNBC | Ⅲ | 474 | June 2016 | ICIs (Anti PD-L1) | MSB0010718C |
| **NCT03498716** | A Study Comparing Atezolizumab (Anti PD-L1 Antibody) In Combination With Adjuvant Anthracycline/Taxane-Based Chemotherapy Versus Chemotherapy Alone In Patients With Operable Triple-Negative Breast Cancer (IMpassion030) | WO39391  2016-003695-47  BIG 16-05  AFT-27  ALEXANDRA | Recruiting  No Results | Stage II-III TNBC | Ⅲ | 2300 | August 2, 2018 | ICIs (Anti PD-L1)  Chemotherapy | Atezolizumab+  Chemotherapy  (Paclitaxel+Doxorubicin/Epirubicin+ Cyclophosphamide) |
| **NCT03197935** | A Study to Investigate Atezolizumab and Chemotherapy Compared With Placebo and Chemotherapy in the Neoadjuvant Setting in Participants With Early Stage Triple Negative Breast Cancer (IMpassion031) | WO39392  2016-004734-22 | Active, not recruiting  Has Results | Early Stage TNBC | Ⅲ | 333 | July 24, 2017 | ICIs (Anti PD-L1)  Chemotherapy | Atezolizumab＋  Chemotherapy  (Nab-paclitaxel+Doxorubicin+Cyclophosphamide+Filgrastim+Pegfilgrastim) |
| **NCT04345913** | Testing the Addition of Copanlisib to Eribulin for the Treatment of Advanced-Stage Triple Negative Breast Cancer | NCI-2020-02319  10382  UM1CA186689  UM1CA1867 | Recruiting  No Results | Advanced-Stage TNBC | I/II | 18 | August 10, 2020 | PI3K inhibitor | Copanlisib |
| **NCT02425891** | A Study of Atezolizumab in Combination With Nab-Paclitaxel Compared With Placebo With Nab-Paclitaxel for Participants With Previously Untreated Metastatic Triple-Negative Breast Cancer (IMpassion130) | WO29522  2014-005490-37 | Completed  Has Result | Previously Untreated Metastatic TNBC | Ⅲ | 902 | June 23, 2015 | ICIs (Anti PD-L1)  Chemotherapy | Atezolizumab＋  Chemotherapy  (Nab-Paclitaxel) |
| **NCT02322814** | A Study of Cobimetinib Plus Paclitaxel, Cobimetinib Plus Atezolizumab Plus Paclitaxel, or Cobimetinib Plus Atezolizumab Plus Nab-Paclitaxel as Initial Treatment for Participants With Triple-Negative Breast Cancer That Has Spread | WO29479  2014-002230-32 | Active, not recruiting  Has Result | Metastatic or locally advanced TNBC | Ⅱ | 169 | November 25, 2014 | MEK inhibitor  ICIs (Anti PD-L1)  Chemotherapy | Cobimetinib+ Atezolizumab+ Chemotherap  (Nab-Paclitaxe/ Paclitaxel) |
| **NCT03316586** | A Phase II Study of Nivolumab in Combination With Cabozantinib for Metastatic Triple-negative Breast Cancer | 17-324 | Completed  No Results | Metastatic TNBC | Ⅱ | 18 | November 30, 2017 | ICIs (Anti PD-L1)  Chemotherapy | Nivoluma+  Chemotherapy  (Cabozantinib) |
| **NCT03546686** | Peri-Operative Ipilimumab+Nivolumab and Cryoablation in Women With Triple-negative Breast Cancer | IIT2018-01-McArthur-IPI | Recruiting  No Results | TNBC | Ⅱ | 80 | November 12, 2019 | Surgery  ICIs  (AntiCTLA-4,  Anti PD-1) | Pre-operative cryoablation  +Ipilimumab +Nivolumab |
| **NCT03639948** | Neoadjuvant Phase II Study of Pembrolizumab And Carboplatin Plus Docetaxel in Triple Negative Breast Cancer | IIT-2017-NeoPACT | Recruiting  No Results | TNBC | Ⅱ | 100 | September 4, 2018 | ICIs (Anti PD-1)  Chemotherapy | Pembrolizumab+Chemotherapy(Carboplatin+Docetaxel+Pegfilgrastim) |
| **NCT03289819** | Neoadjuvant Pembrolizumab(Pbr)/Nab-Paclitaxel Followed by Pbr/Epirubicin/Cyclophosphamide in TNBC | IFG-NIB-01  2016-003102-14  U1111-1188-3915 | Completed  No Results | TNBC | Ⅱ | 53 | March 23, 2018 | ICIs (Anti PD-1)  Chemotherapy | Pembrolizumab+  Chemotherapy  (nab-paclitaxel+Epirubicin+Cyclophosphamide) |
| **NCT03356860** | Safety and Efficacy of Durvalumab Combined to Neoadjuvant Chemotherapy in Localized Luminal B HER2(-) and Triple Negative Breast Cancer. | ONCOGHdC2015_01 | Recruiting  No Results | TNBC | I/Ⅱ | 57 | April 13, 2017 | ICIs (Anti PD-L1)  Chemotherapy | Durvalumab+Chemotherapy  (Paclitaxel+Epirubicin+Cyclophosphamide) |
| **NCT04443348** | Pre-op Pembro + Radiation Therapy in Breast Cancer (P-RAD) | 20-157 | Recruiting  No Results | TNBC | Ⅱ | 120 | December 16, 2020 | Radiation  ICIs (Anti PD-1)  Chemotherapy | Radiation+Pembrolizumab+  Chemotherapy  (Paclitaxel+Carboplatin+Doxorubicin+Cyclophosphamide+Doxorubicin) |
| **NCT04188119** | A Proof of Concept Window Trial of the IMmunological Effects of Aveumab and Aspirin in Triple-Negative Breast Cancer | CFT / sp123  2018-004121-80  017NovCC107 | Not recruiting  No Results | TNBC | Ⅱ | 42 | September 2021 | ICIs (Anti PD-L1) | Avelumab+ Aspirin |
| **NCT02768701** | Study Of Single-dose Cyclophosphamide +Pembrolizumab In Patients With Metastatic Triple Negative Breast Cancer | LCCC 1525 | Active, not recruiting  No Results | Metastatic TNBC | Ⅱ | 40 | October 18, 2016 | ICIs (Anti PD-1)  Chemotherapy | Pembrolizumab+Chemotherapy (Cyclophosphamid) |
| **NCT03121352** | Carboplatin, Nab-Paclitaxel and Pembrolizumab for Metastatic Triple-Negative Breast Cancer | CASE6115 | Active, not recruiting  No Results | Metastatic TNBC | Ⅱ | 30 | May 19, 2017 | ICIs (Anti PD-1)  Chemotherapy | Pembrolizumab+Chemotherapy (Nab-paclitaxel+Carboplatin) |
| **NCT02755272** | A Study of Pembrolizumab With Carboplatin and Gemcitabine in Patients With Metastatic Triple Negative Breast Cancer | BR-076  16-1013 | Recruiting  No Results | Metastatic TNBC | Ⅱ | 87 | May 31, 2016 | ICIs (Anti PD-1)  Chemotherapy | Pembrolizumab+Chemotherapy  (Gemcitabinel+Carboplatin) |
| **NCT03164993** | Atezolizumab Combined With Immunogenic Chemotherapy in Patients With Metastatic Triple-negative Breast Cancer | ML39079_ALICE | Recruiting  No Results | Metastatic TNBC | Ⅱ | 75 | June 1, 2017 | ICIs (Anti PD-L1)  Chemotherapy | Atezolizumab+Chemotherapy  (Cyclophosphamide/Pegylated liposomal doxorubicin  +Paclitaxel) |
| **NCT03206203** | Carboplatin With or Without Atezolizumab in Treating Patients With Stage IV Triple Negative Breast Cance | VICC BRE 15136  NCI-2017-01150 | Active, not recruiting  No Results | Stage IV TNBC | Ⅱ | 106 | August 29, 2017 | ICIs (Anti PD-L1)  Chemotherapy | Carboplatin±  Atezolizumab |
| **NCT03464942** | Stereotactic Radiation and Immunotherapy in Patients With Advanced Triple Negative Breast Cancer | 17/013 | Recruiting  No Results | Advanced TNBC | Ⅱ | 52 | August 1, 2018 | Radiotherapy  ICIs (Anti PD-L1) | SABR+  Atezolizumab |
| **NCT03853707** | Ipatasertib in Combination With Carboplatin, Carboplatin/Paclitaxel, or Capecitabine/Atezolizumab in Treating Patients With Metastatic Triple Negative Breast Cancer | 18496  NCI-2019-00465 | Suspended  No Results | Metastatic TNBC | Ⅰ/Ⅱ | 40 | March 4, 2019 | AKT inhibitoer  Chemotherapy | Ipatasertib+  Carboplatin,+Carboplatin/Paclitaxel, or Capecitabine/Atezolizumab |
| **NCT04408118** | First Line Atezolizumab, Paclitaxel, and Bevacizumab (Avastin®) in mTNBC | MedOPP150  2019-001503-20 | Recruiting  No Results | Metastatic TNBC | Ⅱ | 100 | October 5, 2020 | ICIs (Anti PD-L1)  Chemotherapy | Atezolizumab+Chemotherapy (Bevacizumab+ Paclitaxel) |
| **NCT03167619** | Phase II Multicenter Study of Durvalumab and Olaparib in Platinum tReated Advanced Triple Negative Breast Cancer (DORA) | Pro00080769 | Active, not recruiting  No Results | Advanced TNBC | Ⅱ | 50 | October 4, 2018 | PARPi | Olaparib+Durvalumab |
| **NCT02513472** | Study to Evaluate the Efficacy and Safety of Eribulin Mesylate in Combination With Pembrolizumab in Participants With Metastatic Triple-Negative Breast Cancer (mTNBC) | E7389-M001-218  KEYNOTE-150 | Completed  Has Results | Metastatic TNBC | Ⅰ/Ⅱ | 258 | August 28, 2015 | ICIs (Anti PD-1)  Chemotherapy | Eribulin Mesylate  + Pembrolizumab |
| **NCT02730130** | Study to Assess the Efficacy of Pembrolizumab Plus Radiotherapy in Metastatic Triple Negative Breast Cancer Patients | 16-032 | Active, not recruiting  Has Results | Metastatic TNBC | Ⅱ | 17 | April 2016 | ICIs (Anti PD-1)  Radiotherapy | Pembrolizumab+  Radiotherapy |
| **NCT03036488** | Pembrolizumab for early triple-negative breast cancer | 3475-522 2016-004740-11 173567 MK-3475-522 KEYNOTE-522 | Active, not recruiting  Has Results | TNBC | Ⅱ | 1174 | March 2021 | ICIs (Anti PD-1)  Chemotherapy | Pembrolizumab+  Chemotherapy(Carboplatin+Paclitaxel+Doxorubicin+Epirubicin+Cyclophosphamide） |
| **NCT03872388** | Atorvastatin in Treating Patients With Stage IIb-III Triple Negative Breast Cancer Who Did Not Achieve a Pathologic Complete Response After Receiving Neoadjuvant Chemotherapy | 2018-0550  NCI-2019-00004  P30CA016672 | Recruiting  No Results | StageⅡb-Ⅲ TNBC | II | 80 | January 14, 2019 | Chemoitherapy | Atorvastatin+Capecitabine |
| **NCT03756298** | Efficacy and Safety of Atezolizumab Plus Capecitabine Adjuvant Therapy for Triple Receptor-Negative Breast Cancer | ATOX-2018 | Recruiting  No Results | TNBC | II | 284 | January 15, 2019 | ICIs (Anti PD-L1)  Chemotherapy | Atezolizumab+Capecitabine |
| **NCT03424005** | A Study Evaluating the Efficacy and Safety of Multiple Immunotherapy-Based Treatment Combinations in Patients With Metastatic or Inoperable Locally Advanced Triple-Negative Breast Cancer | CO40115  2017-002038-21 | Recruiting  No Results | TNBC | I/II | 280 | April 2, 2018 | ICIs (Anti PD-L1)  Chemotherapy | Capecitabine, Atezolizumab,Ipatasertib, SGN-LIV1A, Bevacizumab, Chemotherapy (Gemcitabine+Carboplatin or Eribulin), Selicrelumab,Tocilizumab, Nab-Paclitaxel, SG |
| **NCT02996825** | Mirvetuximab Soravtansine and Gemcitabine Hydrochloride in Treating Patients with FRalpha-Positive Recurrent Ovarian, Primary Peritoneal, Fallopian Tube, Endometrial, or Triple Negative Breast Cancer | 16294  NCI-2016-01913 | Recruiting  No Results | TNBC | I | 44 | March 22, 2017 | Chemotherapy  ADC | Gemcitabine+  IMGN853 |
| **NCT02411656** | Pembrolizumab in Treating Patients With Stage IV Metastatic or Recurrent Inflammatory Breast Cancer or Triple- Negative Breast Cancer Who Have Achieved Clinical Response or Stable Disease to Prior Chemotherapy | 2014-0533  NCI-2015-00671 | Recruiting  No Results | TNBC | II | 35 | June 11, 2015 | ICIs (Anti PD-1) | Pembrolizumab |
| **NCT03893955** | A Study to Determine the Safety, Tolerability, Pharmacokinetics, and Preliminary Efficacy of ABBV-927 With ABBV-368, Budigalimab (ABBV-181) and/or Chemotherapy in Participants With Locally Advanced or Metastatic Solid Tumors | M19-037  2019-000478-45 | Recruiting  No Results | TNBC | II | 150 | May 21, 2019 | Combination (Chemotherapy) | ABBV-927+ABBV-368/ABBV-368+ABBV-181/Carboplatin+ABBV-368/Carboplatin+ABBV-181/Carboplatin/Nab-paclitaxel+ABBV-368/ ABBV-368+ABBV-181 |
| **NCT04461600** | A Study of AL101 Monotherapy in Patients With Notch Activated Triple Negative Breast Cancer | AL-TNBC-01 | Recruiting  No Results | TNBC | II | 67 | August 14, 2020 | Inhibitor of gamma secretase-mediated Notch signaling | AL101 |
| **NCT02689427** | Enzalutamide and Paclitaxel Before Surgery in Treating Patients With Stage I-III Androgen Receptor-Positive Triple-Negative Breast Cancer | 2015-0488  NCI-2016-00367  P30CA016672 | Recruiting  No Results | AR+ TNBC | II | 37 | September 22, 2016 | Androgen receptors | Enzalutamide, Paclitaxel |
| **NCT04504916** | A Study of Zilovertamab Vedotin (MK-2140) (VLS-101) in Participants With Solid Tumors (MK-2140-002) | 2140-002  VLS-101-0003  MK-2140-002 | Recruiting  No Results | TNBC | II | 90 | October 7, 2020 | ADC | Zilovertamab vedotin |
| **NCT04770272** | Study to Compare a Mono Atezolizumab Window Followed by a Atezolizumab - CTX Therapy With Atezolizumab - CTX Therapy | Phaon1 | Recruiting  No Results | TNBC | II | 458 | March 1, 2021 | ICIs (Anti PD-L1) | Atezolizumab |
| **NCT03838367** | Leronlimab (PRO 140) Combined With Carboplatin in Patients With CCR5+ mTNBC | CD07_TNBC | Recruiting  No Results | TNBC | I/II | 48 | April 22, 2019 | CCR5 antagonist  Chemotherapy | Leronlimab+  Carboplatin |
| **NCT04429542** | Study of Safety and Tolerability of BCA101 Alone and in Combination With Pembrolizumab in Patients With EGFR- driven Advanced Solid Tumors | BCA101X1101 | Recruiting  No Results | TNBC | I | 292 | June 1, 2020 | ICIs (Anti PD-1) | Pembrolizumab |
| **NCT03808662** | Randomized Study of Stereotactic Body Radiation Therapy (SBRT) in Patients With Oligoprogressive Metastatic Cancers of the Breast and Lung | 18-431 | Recruiting  No Results | TNBC | II | 160 | January 16, 2019 | Radiotherapy | SBRT |
| **NCT05094804** | A Study of OR2805, a Monoclonal Antibody Targeting CD163, Alone and in Combination With a PD-1 Inhibitor | OR2805-101 | Recruiting  No Results | TNBC | I/II | 130 | September 9, 2021 | ICIs (Anti PD-1)  Chemotherapy | OR2805+ Pembrolizumab+ Nivolumab |
| **NCT05001282** | A Study to Evaluate ELU001 in Patients With Solid Tumors That Overexpress Folate Receptor Alpha (FR#) | ELU-FR#-1 | Recruiting  No Results | TNBC | I/II | 166 | September 13, 2021 | ADC | ELU001 |
| **NCT03207867** | A Phase 2 Study of NIR178 in Combination With PDR001 in Patients With Solid Tumors and Non-Hodgkin Lymphoma | CNIR178X2201  2017-000241-49 | Recruiting  No Results | TNBC | II | 376 | August 28, 2017 | A2AR antagonist  MAb | NIR178 + PDR001 |
| **NCT03549000** | A Phase I/Ib Study of NZV930 Alone and in Combination With PDR001 and /or NIR178 in Patients With Advanced Malignancies. | CNZV930X2101  2018-000153-51 | Recruiting  No Results | TNBC | I | 344 | July 18, 2018 | CD73 inhibitor  ICIs | NZV930+ PDR001/ NIR178/ NIR178 and PDR001 |
| **NCT01042379** | I-SPY TRIAL: Neoadjuvant and Personalized Adaptive Novel Agents to Treat Breast Cancer | 097517 | Recruiting  No Results | TNBC | II | 4000 | March 1, 2010 |  |  |
| **NCT04768426** | Serial Circulating Tumor DNA (ctDNA) Monitoring During Adjuvant Capecitabine in Early Triple-negative Breast Cancer | IRB-57723  BRS0121 | Recruiting  No Results | TNBC | II | 25 | February 3, 2021 | Chemotherapy | Capecitabine |
| **NCT04301011** | Study of TBio-6517, Given Intratumorally, Alone or in Combination With Pembrolizumab, in Solid Tumors | TBio-6517-ITu-001 | Recruiting  No Results | TNBC | I/II | 114 | June 2, 2020 | ICIs (Anti PD-1) | TBio-6517、Pembrolizumab |
| **NCT02938442** | Vaccination of Triple Negative Breast Cancer Patients | 206010 | Recruiting  No Results | TNBC | I/II | 102 | January 25, 2019 | Vaccine | P10s-PADR |
| **NCT04115306** | Phase 1/1b Study of Oral PMD-026 in Patients With Metastatic Breast Cancer and Metastatic Triple Negative Breast Cancer | PMD-026-1-001 | Recruiting  No Results | Metastatic TNBC | I | 50 | November 14, 2019 | RSK1-4 inhibitor | PMD-026 |
| **NCT05076682** | Reverse Triple Negative Immune Resistant Breast Cancer | 2107239-9 | Recruiting  No Results | TNBC | II | 20 | October 15, 2021 | ICIs (Anti PD-1)  Chemotherapy | sodium cromoglicate + anti-PD-1 + chemo |
| **NCT04927884** | A Study of Sacituzumab With Chemoimmunotherapy to Treat Advanced Triple-Negative Breast Cancer After Prior Therapies | QUILT-3.058 | Recruiting  No Results | Advanced TNBC | I/II | 79 | September 27, 2021 | ADC (Anti TROP-2)  Chemotherapy | Sacituzumab+Chemoimmunotherapy  (Cyclophosphamide, N-803, and PD-L1 t-haNK) |
| **NCT04060342** | GB1275 Monotherapy and in Combination With an Anti-PD1 Antibody in Patients With Specified Advanced Solid Tumors or in Combination With Standard of Care in Patients With Metastatic Pancreatic Adenocarcinoma | GB1275-1101  (KEYNOTE- A36) | Recruiting  No Results | TNBC | I/II | 242 | August 13, 2019 | Vaccine  Chemotherapy | P10s-PADRE+Doxorubicin+Cyclophosphamide + Paclitaxel |
| **NCT03742102** | A Study of Novel Anti-cancer Agents in Patients With Metastatic Triple Negative Breast Cancer | D933LC00001  2018-000764-29 | Recruiting  No Results | Metastatic TNBC | I/II | 200 | December 21, 2018 | ICIs (Anti PD-l1) | Durvalumab |
| **NCT03454451** | CPI-006 Alone and in Combination With Ciforadenant and With Pembrolizumab for Patients With Advanced Cancers | CPI-006-001 | Recruiting  No Results | TNBC | I | 378 | April 25, 2018 | mAb | CPI-006+ciforadenant/ pembrolizumab |
| **NCT05086692** | A Beta-only IL-2 ImmunoTherapY (ABILITY) Study | MDNA11-01 | Recruiting  No Results | TNBC | I/II | 80 | August 27, 2021 | IL-2-albumin | MDNA11+checkpoint inhibitor |
| **NCT04725331** | A Clinical Trial Assessing BT-001 Alone and in Combination With Pembrolizumab in Metastatic or Advanced Solid Tumors | BT-001.01 | Recruiting  No Results | Metastatic or Advanced TNBC | I/II | 48 | February 25, 2021 | ICIs (Anti PD-1, anti-CTLA4) | BT-001+Pembrolizumab |
| **NCT04577963** | A Study of Fruquintinib in Combination With Tislelizumab in Advanced Triple Negative Breast Cancer | 2020-013-00US3 | Recruiting  No Results | Advanced TNBC | I/II | 72 | June 1, 2021 | TKIs  ICIs (anti PD-1) | Fruquintinib+Tislelizumab |
| **NCT04504669** | First Time in Human Study of AZD8701 With or Without Durvalumab in Participants With Advanced Solid Tumours | D9950C00001  2019-004539-22  04504669 | Recruiting  No Results | Advanced TNBC | I | 123 | August 18, 2020 | FOXP3 antisense oligonucleotide  ICIs (Anti PD-L1) | AZD8701+ Durvalumab |
| **NCT03961698** | Evaluation of IPI-549 Combined With Front- line Treatments in Pts. With Triple-Negative Breast Cancer or Renal Cell Carcinoma (MARIO-3) | IPI-549-03 | Recruiting  No Results | TNBC | II | 90 | December 17, 2019 | PI3K inhibitor  ICIs (anti PD-L1)  Chemotherapy | IPI-549+ Atezolizumab+ nab-paclitaxel/ Bevacizumab |
| **NCT03519178** | A Safety, Pharmacokinetic, Pharmacodynamic and Anti-Tumor Study of PF-06873600 as a Single Agent and in Combination With Endocrine Therapy | C3661001 | Recruiting  No Results | TNBC | II | 160 | March 7, 2018 | CDK inhibitor | PF-06873600 |
| **NCT03554109** | QUILT-3.057: NANT Neoadjuvant Triple- Negative Breast Cancer (TNBC) Vaccine | QUILT-3.057 | Withdrawn  No Results | TNBC | II | 0 | September 2018 | ICIs (Anti PD-L1) | Avelumab |
| **NCT02435680** | Efficacy Study of MCS110 Given With Carboplatin and Gemcitabine in Advanced Triple Negative Breast Cancer (TNBC) | CMCS110Z2201  2015-000179-29 | Completed  Has Results | Advanced TNBC | II | 50 | August 10, 2015 | Anti-M-CSF mAb) | MCS110 |
| **NCT03855358** | A Study of TQB2450 Injection and Anlotinib Hydrochloride Capsules to Treat Triple Negative Breast Cancer (TNBC) | TQB2450-Ib-07 | Unknown status  No Results | TNBC | I | 30 | May 9, 2019 | ICIs(anti-PD-L1)  Chemotherapy | TQB2450  Anlotinib Hydrochioride |
| **NCT03057600** | Study of CB-839 in Combination w/ Paclitaxel in Patients of African Ancestry and Non-African Ancestry With Advanced Triple Negative Breast Cancer (TNBC) | CX-839-007 | Completed  No Results | Advanced TNBC | II | 52 | May 15, 2017 | Glutaminase inhibitor  Chemotherapy | CB-839+ Paclitaxel |
| **NCT02482311** | Safety, Tolerance, PK, and Anti-tumour Activity of AZD1775 Monotherapy in Patients With Advanced Solid Tumours | D6015C00001  REFMAL 383 | Completed  No Results | Advanced TNBC | I | 92 | July 1, 2015 | Wee1 inhibitor | AZD 1775 |
| **NCT03184558** | Bemcentinib (BGB324) in Combination With Pembrolizumab in Patients With TNBC | BGBC007  MK-3475 PN530  2016-003608-30 | Completed  No Results | TNBC | II | 29 | July 26, 2017 | TKIs (AXL inhibitor)  ICIs (Anti PD-1) | Bemcentinib; pembrolizumab |
| **NCT03045393** | Mirvetuximab Soravtansine (IMG853) in Folate Receptor Alpha-expressing TNBC | Pro00074621 | Withdrawn  No Results | TNBC | I | 0 | April 17, 2017 | ADC (target FRα) | IMGN853 |
| **NCT03719326** | A Study to Evaluate Safety/Tolerability of Immunotherapy Combinations in Participants With Triple-Negative Breast Cancer or Gynecologic Malignancies | AB928CSP0002 | Completed  No Results | TNBC | I | 35 | October 15, 2018 | A2aR and A2bR antagonist  Chemotherapy | Etrumadenant+ PLD/ NP/(IPI-549+ PLD) |
| **NCT01884285** | AZD8186 First Time In Patient Ascending Dose Study | D4620C00001 | Completed  No Results | TNBC | I | 147 | July 9, 2013 | PI3K inhibitor  mTOR inhibitor | AZD8186, AZD2014 |
| **NCT04067102** | Nab-paclitaxel Based Regimens VS Paclitaxel Based Regimens in Neoadjuvant Treatment for TNBC | TNBC-NEO | Withdrawn  No Results | TNBC | --- | 0 | May 10, 2019 | Chemotherapy | nab-paclitaxel |
| **NCT02622074** | Safety and Efficacy Study of Pembrolizumab (MK-3475) in Combination With Chemotherapy as Neoadjuvant Treatment for Participants With Triple Negative Breast Cancer (TNBC) (MK-3475-173/ KEYNOTE-173) | 3475-173  2015-002405-11  MK-3475-173  KEYNOTE-173 | Completed  Has Results | TNBC | I | 60 | January 27, 2016 | ICIs (Anti PD-1)  Chemotherapy | Pembrolizumab+ Chemotherapy |
| **NCT02531425** | Evaluation of Pharmacodynamic Effects of IT-pIL12-EP in Patients With TNBC | OMS-I140 | Completed  No Results | TNBC | I | 10 | September 2015 | IT-pIL12-EP | IT-pIL12-EP |
| **NCT03289819** | Neoadjuvant Pembrolizumab(Pbr)/Nab-Paclitaxel Followed by Pbr/Epirubicin/Cyclophosphamide in TNBC | IFG-NIB-01  2016-003102-14  U1111-1188-3915 | Completed  No Results | TNBC | II | 53 | March 23, 2018 | ICIs (Anti PD-1)  Chemotherapy | Pembrolizumab+nab-paclitaxel+Epirubicin+Cyclophosphamide |
| **NCT02353988** | AR-inhibitor Bicalutamide in Treating Patients With TNBC | JinlingH-001 | Unknown status  No Results | AR+ TNBC | II | 60 | January 2015 | AR inhibitor | Bicalutamide |
| **NCT02402764** | Phase 2 Trial of Selinexor (KPT-330) for Metastatic Triple Negative Breast Cancer (TNBC) | Other Ids:  MCC-18150 | Completed  Has Results | TNBC | II | 10 | July 8, 2015 | SINE | Selinexor |
| **NCT01920061** | A Study Of PF-05212384 In Combination With Other Anti-Tumor Agents and in Combination With Cisplatin in Patients With Triple Negative Breast Cancer in an Expansion Arm (TNBC) | B2151002  2013-001390-24 | Completed  Has Results | TNBC | I | 110 | September 10, 2013 | PI3K/mTOR inhibitor  Chemotherapy | PF-05212384+Docetaxel+ Cisplatin+ Dacomitinib |
| **NCT03945604** | A Trial of SHR-1210 (an Anti-PD-1 Inhibitor) in Combination With Apatinib and Fluzoparib in Patients With TNBC | FZPL-Ib-105 | Unknown status  No Results | TNBC | I | 52 | June 4, 2019 | ICIs (Anti PD-1)  TKIs (VEGF)  PARPi | SHR-1210+Apatinib +Fluzoparib |
| **NCT03394287** | A Trial of SHR-1210 (an Anti-PD-1 Antibody) in Combination With Apatinib in Patients With Advanced TNBC | SHR-1210-APTN-IIT-TNBC | Completed  No Results | TNBC | II | 40 | January 10, 2018 | ICIs (Anti PD-1)  TKIs (VEGF) | SHR-1210+ Apatinib |
| **NCT01378533** | The Trial Comparing Dose-dense AC-T With PC as Adjuvant Therapy for TNBC | CH-BC-012 | Unknown status  No Results | TNBC | III | 100 | May 2011 | Chemotherapy | epirubicin,cyclophosphamide, paclitaxel, carboplatin, G-CSF |
| **NCT02641847** | TA(E)C-GP Versus A(E)C-T for the High Risk TNBC Patients and Validation of the mRNA-lncRNA Signature | 1506147-4 | Unknown status  No Results | TNBC | II/III | 503 | July 2015 | Chemotherapy | Docetaxel+doxorubicin /epirubicin+cyclophosphamide+ gemcitabine_+_cisplatin |
| **NCT03251313** | PD-1(Programmed Death-1) Antibody +GP as First Line Treatment for Triple Negative Breast Cancer (TNBC) Patients | Fudan-P1-201701 | Unknown status  No Results | TNBC | I | 33 | November 2019 | ICIs (Anti PD-1) | JS001+GP |
| **NCT02511847** | Trial of Afatinib With Paclitaxel for Neoadjuvant Therapy of TNBC and Research of Biomarkers of Afatinib | 201408024MIPC | Unknown status  No Results | TNBC | II | 40 | August 2015 | TKIs (EGFR inhibitor) | Afatinib |
| **NCT02341911** | Gemcitabine Plus Cisplatin Versus Gemcitabine Plus Carboplatin in Triple Negative Breast Cancer (TNBC) | Fudan BR2015-16 | Unknown status  No Results | TNBC | II | 150 | January 2015 | Chemotherapy | Gemcitabine+cisplatin/carboplatin |
| **NCT01287624** | Gemcitabine Plus Cisplatin Versus Gemcitabine Plus Paclitaxel in Triple Negative Breast Cancer (TNBC) | Fudan BR2010-04  CBCSG 006 | Completed  No Results | TNBC | III | 240 | January 2011 | Chemotherapy | Gemcitabine+cisplatin/Paclitaxel |
| **NCT01639248** | Phase 2 Study - Aurora + Angiogenic Kinase Inhibitor ENMD-2076 in Previously Treated Locally Advanced + Metastatic TNBC | 2076-CL-005 | Completed  No Results | Previously Treated Locally Advanced or Metastatic TNBC | II | 41 | July 2012 | FLT3 inhibitor | ENMD-2076 |
| **NCT01953536** | Safety and Efficacy Study of Vintafolide and Vintafolide Plus Paclitaxel Compared to Paclitaxel Alone in Participants With Triple Negative Breast Cancer (TNBC) (MK-8109-004) | 8109-004  2012-005170-65 | Withdrawn  No Results | TNBC | II | 0 | April 2014 | Chemotherapy | Vintafolide+Etarfolatide+Folic acid+ (Paclitaxel+ Paclitaxel) |
| **NCT01333423** | Maximum Tolerated Dose (MTD) of Liposomal Doxorubicin in Combination With Seliciclib for Patients With Metastatic Triple Negative Breast Cancer (TNBC) | 2011-0013  1R01CA152228-01A1 | Withdrawn  No Results | Metastatic TNBC | I | 0 | September 2012 | CDK inhibitor  Chemotherapy | Liposomal Doxorubicin +Seliciclib |
| **NCT03332368** | Clinical Study on Triple Negative Breast Cancer With Chinese Medicine | LonghuaH(TNBC) Special Research on TCM | Unknown status  No Results | TNBC | --- | 620 | January 1, 2017 | Traditional Chinese medicine | Traditional Chinese medicine |
| **NCT03872505** | Pre-operAtive Non-Anthracycline Chemotherapy, Durvalumab +/- RAdiation Therapy in Triple Negative Breast Cancer | IIT2018-17-McArthur-  TCDRT | Withdrawn  No Results | TNBC | II | 0 | July 2022 | Chemotherapy  ICIs (anti PD-L1)  Radiotherapy | Non-Anthracycline, Durvalumab +/- RAdiation |
| **NCT02981303** | Study of Imprime PGG and Pembrolizumab in Advanced Melanoma and Triple Negative Breast Cancer | BT-CL-PGG-MEL/  BCA-1621/MK3475 | Completed  No Results | \TNBC | II | 64 | February 22, 2017 | Chemotherapy  ICIs (anti PD-L1)  Radiotherapy | Chemotherapy+ Durvalumab) + Radiation Therapy |
| **NCT02719691** | Phase I Study of MLN0128 and MLN8237 in Patients With Advanced Solid Tumors and Metastatic Triple-negative Breast Cancer | 15-1135.cc | Completed  No Results | Metastatic TNBC | I | 49 | May 13, 2016 | mTOR inhibitor  Aurora A inhibit | Alisertib、MLN0128 |
| **NCT03106077** | Mirvetuximab Soravtansine as First Line in Treating Patients With Triple Negative Breast Cancer | 2016-0683  NCI-2018-01213 | Completed  Has Results | TNBC | II | 96 | June 5, 2017 | ADC (targeted FRα) | Mirvetuximab Soravtansine |
| **NCT02032277** | A Study Evaluating Safety and Efficacy of the Addition of ABT-888 Plus Carboplatin Versus the Addition of Carboplatin to Standard Chemotherapy Versus Standard Chemotherapy in Subjects With Early Stage Triple Negative Breast Cancer | M14-011  2013-002377-21 | Completed  No Results | TNBC | III | 634 | April 2, 2014 | ADC (targeted FRα) | Mirvetuximab Soravtansine |
| **NCT01204125** | Two Regimens of SAR240550/Weekly Paclitaxel and Paclitaxel Alone as Neoadjuvant Therapy in Triple Negative Breast Cancer Patients | TCD11419  2010-018960-17 | Completed  No Results | TNBC | II | 141 | September 2010 | Chemotherapy  PARPi | Paclitaxel +Iniparib |
| **NCT02723877** | PQR309 and Eribulin in Metastatic HER2 Negative and Triple-negative Breast Cancer (PIQHASSO) | PQR309-007 | Completed  No Results | Metastatic TNBC | I/II | 41 | March 28, 2016 | PI3K inhibitor | PQR309+ Eribulin |
| **NCT02593227** | Folate Receptor Alpha Peptide Vaccine With GM-CSF in Patients With Triple Negative Breast Cancer | FRV-002 | Completed  No Results | TNBC | II | 80 | April 2016 | Chemotherapy | Cyclophosphamide |
| **NCT02413320** | Neoadjuvant Study of Two Platinum Regimens in Triple Negative Breast Cancer | 2015-IIT-Neoadjuvant-  BRST-TNBC | Completed  Has Results | TNBC | II | 101 | July 2015 | Chemotherapy | Paclitaxe+Paclitaxel+Doxorubicin+ Cyclophosphamide |
| **NCT04541420** | Eribulin in mTNBC Patients | YOUNGBC-12 | Completed  No Results | Breast Cancer |  | 208 | December 15, 2019 | Chemotherapy | Eribulin |
| **NCT02307240** | Open Label, Multi-center Study to Assess the Safety, Tolerability and Pharmacokinetics of CUDC-907 in Subjects With Advanced/Relapsed Solid Tumors | CUDC-907-102 | Completed  No Results | TNBC | I | 43 | November 2014 | HDAC inhibitor | CUDC-907 |
| **NCT03577743** | Effect of Bevacizumab in Metastatic Triple Negative Breast Cancer | BMTN | Completed  No Results | MetastaticTNBC | II | 54 | July 1, 2018 | anti-VEGF | Bevacizumab |
| **NCT02178722** | Study to Explore the Safety, Tolerability and Efficacy of MK-3475 in Combination With INCB024360 in Participants With Selected Cancers | INCB 24360-202/  ECHO-202 | Completed  Has Results | TNBC | I/II | 444 | July 17, 2014 | ICIs (Anti PD-1)  IDO1 inhibitor | MK-3475+ INCB024360 |
| **NCT03098550** | A Study to Test the Safety and Effectiveness of Nivolumab Combined With Daratumumab in Patients With Pancreatic, Non-Small Cell Lung or Triple Negative Breast Cancers, That Have Advanced or Have Spread | CA209-9G  2017-000367-33 | Completed  Has Results | Advanced TNBC | I/II | 105 | June 15, 2017 | ICIs (Anti PD-1)  Anti CD38 | Nivolumab+ Daratumumab |
| **NCT02012634** | Metronomic Chemotherapy of Capecitabine After Standard Adjuvant Chemotherapy in Operable Triple Negative Breast Cancer | TPYS-01 | Unknown status  No Results | TNBC | -- | 340 | January 2013 | Chemotherapy | Capecitabine |
| **NCT03394027** | ONC201 in Recurrent/Refractory Metastatic Breast Cancer and Advanced Endometrial Carcinoma | 180034  18-C-0034 | Completed  No Results | TNBC | II | 30 | January 17, 2018 | DRD2 inhibitor | ONC201 |
| **NCT01938638** | Open Label Phase I Dose Escalation Study With BAY1143572 in Patients With Advanced Cancer | 16519 | Completed  No Results | TNBC | I | 80 | September 26, 2013 | CDK inhibitor | BAY1143572 |
| **NCT01467310** | Defining the Triple Negative Breast Cancer Kinome Response to GSK1120212 | LCCC 1122  11-1689  CA058223 | Completed  No Results | TNBC | I | 15 | January 2012 | MEK inhibito | GSK1120212 |
| **NCT00998036** | Study of Temsirolimus, Erlotinib and Cisplatin in Solid Tumors | AAAD8279 | Completed  No Results | TNBC | I | 9 | September 2009 | mTOR inhibitor  Chemotherapy | Temsirolimus+Cisplatin+Erlotinib |
| **NCT01307891** | Abraxane With or Without Tigatuzumab in Patients With Metastatic, Triple Negative Breast Cancer | F101004001 (UAB1028)  TBCRC 019 | Completed  Has Results | Metastatic TNBC | II | 64 | March 2011 | Chemotherapy  Death receptor-5 agonist | Abraxane+ Tigatuzumab |
| **NCT02900664** | A Study of PDR001 in Combination With CJM112, EGF816, Ilaris® (Canakinumab) or Mekinist® (Trametinib) | CPDR001X2103  2016-000633-49 | Completed  No Results | TNBC | I | 289 | August 23, 2016 | ICIs (anti PD-1) | PDR001+ ACZ885/ CJM112/ TMT212/ EGF816 |
| **NCT01997333** | Study of Glembatumumab Vedotin (CDX-011) in Patients With Metastatic, pNMB Over-Expressing, Triple Negative Breast Cancer | CDX011-04 | Completed  Has Results | Metastatic TNBC | II | 327 | November 2013 | Chemotherapy  ADC | Capecitabine、CDX-011 |
| **NCT01770353** | MM-398 (Nanoliposomal Irinotecan, Nal- IRI) to Determine Tumor Levels and to Evaluate the Feasibility of Ferumoxytol Magnetic Resonance Imaging to Measure Tumor Associated Macrophages and to Predict Patient Response to Treatment | MM-398-01-01-02 | Completed  Has Results | TNBC | I | 45 | November 2012 | Chemotherapy | Ferumoxytol+ MM-398 |

(Clinicaltrials.gov, accessed on November 1, 2021)

Num. *: number of patients

ADC: Antibody-Drug Conjugates; AR: Androgen Receptor; AR+: Androgen Receptor Positive; BET: Bromodomain and Extra-Terminal; CDKs: Cyclin-Dependent Kinases; DRD2: Dopamine Receptor D2; EGFR: Epidermal Growth Factor Receptor;

FGFR: Fibroblast Growth Factor Receptor; HDAC: Histone Deacetylase; HyFc: Hybrid Fc; IAP: Inhibitor of Apoptosis; ICIs: Immune Checkpoint Inhibitors; IDO1: Indoleamine-(2,3)-Dioxygenase; IGFR: Insulin-like Growth Factor Receptor;

IT-pIL12-EP: Interleukin-2 (IL-2)-Albumin; MNK1/2: MAP kinase-Interacting Serine/threonine Kinases 1 and 2; mAb: Monoclonal Antibody; PARPi: Poly-(ADP)-Ribose Polymerase; RTKs: Receptor Tyrosine Kinases;

SABR: Stereotactic Ablative Body Radiotherapy; SBRT: Randomized Study of Stereotactic Body Radiation Therapy; SINE: Selective Inhibitor of Nuclear Export; SG: sacituzumab govitecan; TKIs: Tyrosine Kinase Inhibitors;

TNBC: Triple‑Negative Breast Cancer; VBIR-2: Vaccine-Based Immunotherapy Regimen-2; VEGFR: Vascular Endothelial Growth Factor Receptor
